# Reprogramming M2 macrophages via TLR7/8 agonist-loaded hollow MnO_2_ nanovehicles to suppress the progression of liver cancer

**DOI:** 10.1371/journal.pone.0358567

**Published:** 2026-09-18

**Authors:** Pei-pei He, Zheng-ju Hou, Han-mei Li, Ji-xuan Zhang, An-Qi Chen, Quan-you Miao, Chao-Feng Yang, Jin-hong Yu, Yang Li

**Affiliations:** 1 Department of Radiology, Afﬁliated Hospital of North Sichuan Medical College, Nanchong, Sichuan, China; 2 Innovation Centre for Science and Technology of North Sichuan Medical College, Nanchong, Sichuan, China; 3 Biotechnology Innovation Drug Application and Transformation Key Laboratory of Sichuan Province, North Sichuan Medical College, Nanchong, Sichuan Province, China; 4 Department of Ultrasound, Afﬁliated Hospital of North Sichuan Medical College, Nanchong, Sichuan, China; Universidade Federal do Rio de Janeiro, BRAZIL

## Abstract

As a prevalent malignancy, liver cancer ranks as the third most common cause of cancer mortality globally. Within the tumor microenvironment, tumor-associated macrophages (TAMs) exhibit contrasting functional phenotypes: the anti-tumor M1 type and the pro-tumor M2 type. Reprogramming TAMs from the M2 to the M1 phenotype has emerged as a promising immunotherapeutic strategy. Here, we engineered a multi-nano therapeutic system, PAH@HMMDN@R848, by loading a TLR7/8 agonist (R848) onto hollow MnO_2_ nanoparticles (HMMDN) and coating with polyallylamine hydrochloride (PAH). In vitro, this system efficiently repolarized M2 macrophages to the M1 phenotype, evidenced by decreased CD206, Arg-1, IL-10 and increased CD80, iNOS, TNF-α (P < 0.05). Under acidic/glutathione-rich conditions, HMMDN degraded to release Mn^2+^, which served as a T_1_-weighted MRI contrast agent with a high relaxivity (r_1_ = 5.255 mM^-^^1^ s ^-^^1^). Mechanistically, PAH@HMMDN@R848 activated both NF-κB (via R848) and STING (via Mn^2+^) pathways, leading to synergistic M1 polarization. In a subcutaneous H22 liver cancer mouse model, intratumoral injection of PAH@HMMDN@R848 significantly suppressed tumor growth (tumor inhibition rate 80.6%) without obvious systemic toxicity. Immunohistochemistry and cytokine analysis revealed that the treatment increased M1 macrophage infiltration (CD80^+^) and reduced M2 macrophages (CD206^+^) within tumors, along with elevated TNF-α/iNOS and decreased IL-10/Arg-1. This study presents a multifunctional nanoplatform capable of TAMs reprogramming and MRI diagnosis, offering a promising approach for liver cancer theranostics.

## 1. Introduction

Liver cancer represents a malignancy characterized by significant global prevalence and associated mortality. Epidemiological data, collated in 2022, recorded approximately 865,269 new diagnoses and 757,948 fatalities attributed to liver cancer worldwide, establishing this condition as the sixth most frequently diagnosed malignant tumor and the third most common cause of cancer death worldwide [1]. Notably, the burden of liver cancer disproportionately affects China, which contributes 54.3% of the global incidence and 47.1% of mortality, ranking highest internationally [2]. Within the Chinese population, liver cancer manifests primarily as three distinct pathological subtypes: liver cancer, intrahepatic cholangiocarcinoma (ICC), and combined hepatocellular-cholangiocarcinoma (cHCC-CCA). Of these, liver cancer constitutes the predominant form, representing approximately 75–85% of cases [3]. A major challenge lies in the frequent late-stage diagnosis of liver cancer; factors including advanced disease presentation upon initial detection, high rates of therapeutic resistance, and elevated recurrence contribute to a poor prognosis. Consequently, up to 70% of patients with liver cancer are already in intermediate or advanced stages when first diagnosed [4]. The prevention and effective management of liver cancer thus persist as a critical global health challenge.

The tumor microenvironment (TME), comprising the tumor stroma, constitutes a highly dynamic and intricate network that plays a critical role in tumor initiation, progression, and clinical outcomes. This network exerts profound influence over tumorigenesis and development through diverse cellular components and molecular signaling pathways, characterized by its inherent complexity, heterogeneity, and adaptability [5]. Within the TME of liver cancer, cellular constituents encompass tumor-associated macrophages (TAMs), activated hepatic stellate cells (HSCs), and cancer-associated fibroblasts (CAFs), while non-cellular elements include the extracellular matrix (ECM), soluble regulatory molecules, extracellular vesicles, and additional environmental cues [6]. Extensive interactions exist among these diverse TME components. Previous research has demonstrated that TAMs facilitate tumor recurrence and metastasis via multiple mechanisms: stimulating angiogenesis in liver cancer, remodeling the composition of tumor ECM and basement membranes, thus inducing epithelial-mesenchymal transition (EMT), and promoting the emergence and persistence of cancer stem cell traits [7]. HSCs are known to mediate bidirectional communication with cancer cells by secreting various growth factors, proteolytic enzymes, and cytokines; moreover, HSCs play a pivotal bridging role in the transition from liver fibrosis to carcinoma by modulating ECM synthesis and functional expression [8]. CAFs contribute directly to tumor progression by engaging in paracrine signaling with malignant cells and other stromal components, or by structurally remodeling the ECM, thereby fostering an environment conducive to tumor growth, invasion, and dissemination [9]. Furthermore, degradation of the ECM and basement membranes can trigger the expression of specific proteases, which subsequently facilitate tumor cell invasion and metastatic spread [10].

**Fig 1:**
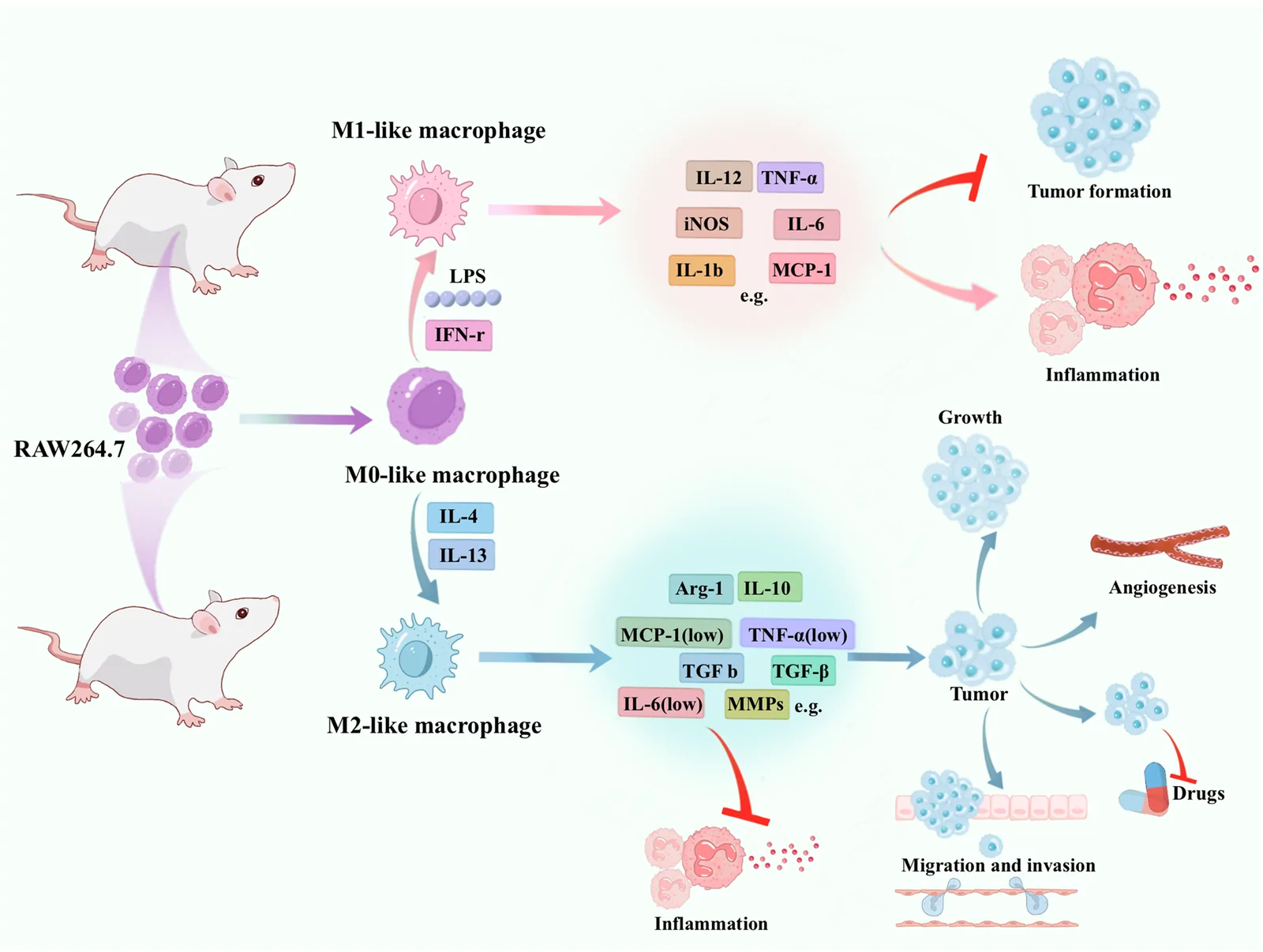
Schematic diagram showing the classification of macrophages.

TAMs, serving as pivotal and multifaceted regulators within the TME, are broadly categorized into two phenotypes: the pro-inflammatory, anti-tumor M1 type and the pro-tumor, anti-inflammatory M2 type [11]. M1-polarized macrophages exert cytotoxic effects on malignant cells by secreting pro-inflammatory cytokines (e.g., tumor necrosis factor-α) within the TME, thus enhancing the recruitment or activation of molecules such as interferon-gamma (IFN-γ) and interleukin (IL)-12, and facilitating the infiltration of immune effector cells [12]. Conversely, M2-polarized macrophages promote primary tumor progression and dissemination through mechanisms that include basement membrane disruption *via* cytokines such as IL-10, IL-13, CCL9, and transforming growth factor, thus fostering neovascularization, and recruiting immunosuppressive cells (Fig 1) [13,14]. Consequently, reprogramming TAMs from the M2 to the M1 phenotype has emerged as a compelling immunotherapeutic approach. This strategy aims to suppress tumor growth by augmenting the secretion of immunostimulatory mediators while diminishing immunosuppressive factors [15]. Previous research indicated that Toll-like receptors (TLRs), owing to their abundant expression on immune and various malignant cells, represent a prevalent target for immunomodulation that are capable of inducing the conversion of M2 TAMs towards the M1 state [16–18].

Resiquimod (R848), a potent dual agonist targeting TLR7 and TLR8, is an Food and Drug Administration (FDA)-sanctioned immunomodulatory agent that activates the NF-κB signaling pathway, leading to IFN-γ production and reduced IL-4 generation. R848 also enhances antibody-dependent cellular phagocytosis (ADCP) and reprograms M2 TAMs towards an M1 phenotype, collectively contributing to its anti-tumor efficacy [19,20]. However, the limited aqueous solubility of R848 restricts its clinical utility [21]. However, polyallylamine hydrochloride (PAH), a weakly basic cationic polymer known for its stability and biocompatibility, can complex with R848 *via* electron transfer and electrostatic interactions, enhancing the surface activity and solubility of the compound. Within the acidic TME, increased protonation of PAH facilitates targeted drug release, thus elevating local therapeutic concentrations [22].

Manganese-based nanomaterials, when integrated with diverse therapeutic modalities, have shown promise in overcoming the limitations of monotherapy, generating synergistic outcomes. The inherent magnetic properties of manganese-based nanomaterials can augment anti-tumor potency or diagnostic precision [23]. Notably, Mn^^2+^^ ions can activate the cGAS-STING signaling pathway, a crucial innate immune pathway that senses cytosolic DNA and triggers type I interferon production, thereby promoting dendritic cell maturation and antigen presentation to T cells [24,25]. The combination of Mn^2+^ with immunotherapy has advanced to Phase I clinical trials, demonstrating favorable prospects for patients with advanced malignancies [26]. Hollow manganese dioxide nanoparticles (HMMDN) represent a prominent class of nanomaterials and respond to acidic pH by decomposing into oxygen and Mn^^2+^^ ions, catalytically consuming intra-tumoral H_2_O_2_ and glucose, and oxidizing glutathione (GSH) to its disulfide form (GSSG) [27]. This activity enhances T1-weighted magnetic resonance imaging (T1WI MRI) under pH/redox conditions [27]. Simultaneously, HMMDN remodels the immunosuppressive TME by attenuating hypoxia-driven TAMs infiltration, stimulating macrophage polarization towards the M1 phenotype, and increasing intra-tumoral CD8^+^ cytotoxic T lymphocyte populations [28]. Therefore, employing HMMDN for R848 delivery not only enables MRI but also provides Mn^2+^ to activate the STING pathway, potentially synergizing with R848-induced NF-κB activation for enhanced TAMs reprogramming.

Based on the above research background and design concept, this study aims to successfully construct a novel multifunctional nanotherapeutic platform—PAH@HMMDN@R848. This platform integrates the immune agonist R848, the TME-responsive carrier HMMDN, and the solubility enhancer PAH. It can improve the delivery efficiency of R848 through PAH, achieve drug release at the tumor site using HMMDN, and generate synergistic effects through the TLR-NF-κB pathway activated by R848 and the STING pathway activated by Mn²^+^, thereby potently driving the repolarization of M2-type macrophages toward the M1 phenotype and effectively inhibiting liver cancer growth both in vitro and in vivo. Simultaneously, this system possesses the potential to serve as a T_1_-weighted MRI contrast agent, offering new possibilities for the integration of diagnosis and treatment in liver cancer.

## 2. Materials and methods

### 2.1. Materials

Chemicals and reagents: Ethanol, ammonium hydroxide (NH_3_·H_2_O), paraformaldehyde (POM), and sodium carbonate (Na_2_CO_3_) were sourced from Sinopharm Chemical Reagent Co., Ltd. (Shanghai, China). Phosphate buffered saline (PBS) was obtained from Gene Biomedical Technology Co., Ltd. (Zhejiang, China). R848, lipopolysaccharide (LPS), and interferon-gamma (IFN-γ) were purchased from MedChemExpress (MCE, Shanghai, China). Enzyme-linked immunosorbent assay (ELISA) kits were obtained from ELK Biotechnology (Wuhan, China). Cell Counting Kit-8 (CCK-8) and Triton X-100 were acquired from Beyotime Biotechnology Co., Ltd. (Shanghai, China). Bovine serum albumin (BSA) was purchased from Biofroxx (Guangzhou, China). 4',6-Diamidino-2-phenylindole (DAPI) was obtained from Sigma-Aldrich (Shanghai, China). Tetraethyl orthosilicate (TEOS) was purchased from Tokyo Chemical Industry Co., Ltd. (TCI, Shanghai, China). Potassium permanganate (KMnO_4_) was acquired from Chongqing Chuandong Chemical Group (Chongqing, China). Polyallylamine hydrochloride (PAH) was sourced from Adamas-beta (Shanghai, China). N,N-Dimethylformamide (DMF) was obtained from Chengdu Kelong Chemical Co., Ltd. (Chengdu, China). The Annexin V-FITC apoptosis detection kit was purchased from BD Biosciences (Shanghai, China). Penicillin-streptomycin solution and cell culture plates (6-well, 24-well, 96-well) were obtained from Yeasen Biotechnology Co., Ltd. (Shanghai, China). Centrifuge tubes (1.5 mL, 2.0 mL, 15 mL) were purchased from Beijing Lanjieke Technology Co., Ltd. (Beijing, China). The DAB substrate kit was obtained from Changdao Biotechnology Co., Ltd. (Shanghai, China).

Antibodies: Polyclonal antibodies against CD80 (A16039) were sourced from Abclonal Biotechnology Co., Ltd. (Wuhan, China); CD206 (sc-70585) from Santa Cruz Biotechnology (Beijing, China); inducible nitric oxide synthase (iNOS, ab178945) from Abcam (Shanghai, China); arginase-1 (Arg-1, bsm-56207R) from Bioss Antibodies (Beijing, China). For signaling pathway analysis, antibodies against NF-κB p65 (#BF8005), phospho-p65 (#3033), IκBα (#9242), phospho-IκBα (#71278), TANK-binding kinase 1 (TBK1, #DF7026), phospho-TBK1 (#AF8190), interferon regulatory factor 3 (IRF3, #DF6895), and phospho-IRF3 (#AF2436) were purchased from Affbiotech (Jiangsu, China). Glyceraldehyde-3-phosphate dehydrogenase (GAPDH, ab9485) was obtained from Abcam (Shanghai, China). Secondary antibodies included CoraLite488-labeled goat anti-rabbit IgG (SA0013−2) from Sanying Biotechnology Co., Ltd. (Wuhan, China) and CY3-labeled goat anti-mouse IgG (AS1111) from Asbury Biotechnology Co., Ltd. (Wuhan, China).

### 2.2. Preparation of nanoparticles

#### 2.2.1. Synthesis of HMMDN.

Initially, a round-bottomed flask was filled with 50 mL of ethanol and 2.5 mL of double-distilled water (ddH_2_O), followed by uniform mixing at 400 g. Subsequently, 250 μL of TEOS was introduced into the flask, and the mixture was stirred at room temperature for 30 min. After this period, 2.5 mL of NH_3_·H_2_O was added dropwise, and stirring continued for an additional 60 min. The resulting mixture was then centrifuged using a high-speed centrifuge (5804R, Eppendorf AG, Hamburg, Germany) at 12,000 × g for 8 min to isolate the SiO_2_ nanoparticle precipitate. This precipitate underwent two ethanol washing cycles, each involving centrifugation at 16,000 × g for 5 min. Finally, the purified product was dispersed in 4 mL of ddH_2_O for storage.

Next, 2 mL of the prepared SiO_2_ solution was combined with 8 mL of ddH_2_O and homogenized *via* ultrasonication using an ultrasonic cell disruptor (SM-1000A, Nanjing Shunma Instrument Equipment Co., Ltd., Nanjing, China). While maintaining sonication, 2 mL of KmnO_4_ solution (3.75 mg/mL) was added dropwise. Ultrasonic treatment proceeded for 60 min, yielding a reddish-brown solution. This mixture was then transferred to an electric heating mantle using a magnetic stirring heating mantle (ZNCL-T, Shanghai Yingyu Industrial Co., Ltd., Shanghai, China) and reacted for 5 h under controlled conditions (external temperature: 40 °C; stirring speed: 400 × g). At the end of the reaction, centrifugation was performed (12,000 × g, 10 min) to generate the SiO_2_@MnO_2_ nanoparticle precipitate. After two washes with ddH_2_O, the final product was suspended in 8 mL of ddH_2_O.

Subsequently, 2060 mg of Na_2_CO_3_ was dissolved in 8 mL of ddH_2_O. To this solution, 2 mL of the SiO_2_@MnO_2_ nanoparticle suspension was added. The reaction proceeded at 60 °C with stirring (400 × g) for 24 h. Centrifugation at 12,000 × g for 10 min isolated the HMMDN nanoparticle precipitate. Following two ddH_2_O washes, the product was dissolved in 3 mL of ddH_2_O. This solution was dialyzed (molecular weight cutoff: 1000 kDa) against ddH_2_O for 12 h to eliminate residual Na_2_CO_3_. The dialyzed product was then adjusted to a final volume of 5 mL with ddH_2_O.

#### 2.2.2. Synthesis of PAH@HMMDN@R848.

**Fig 2:**
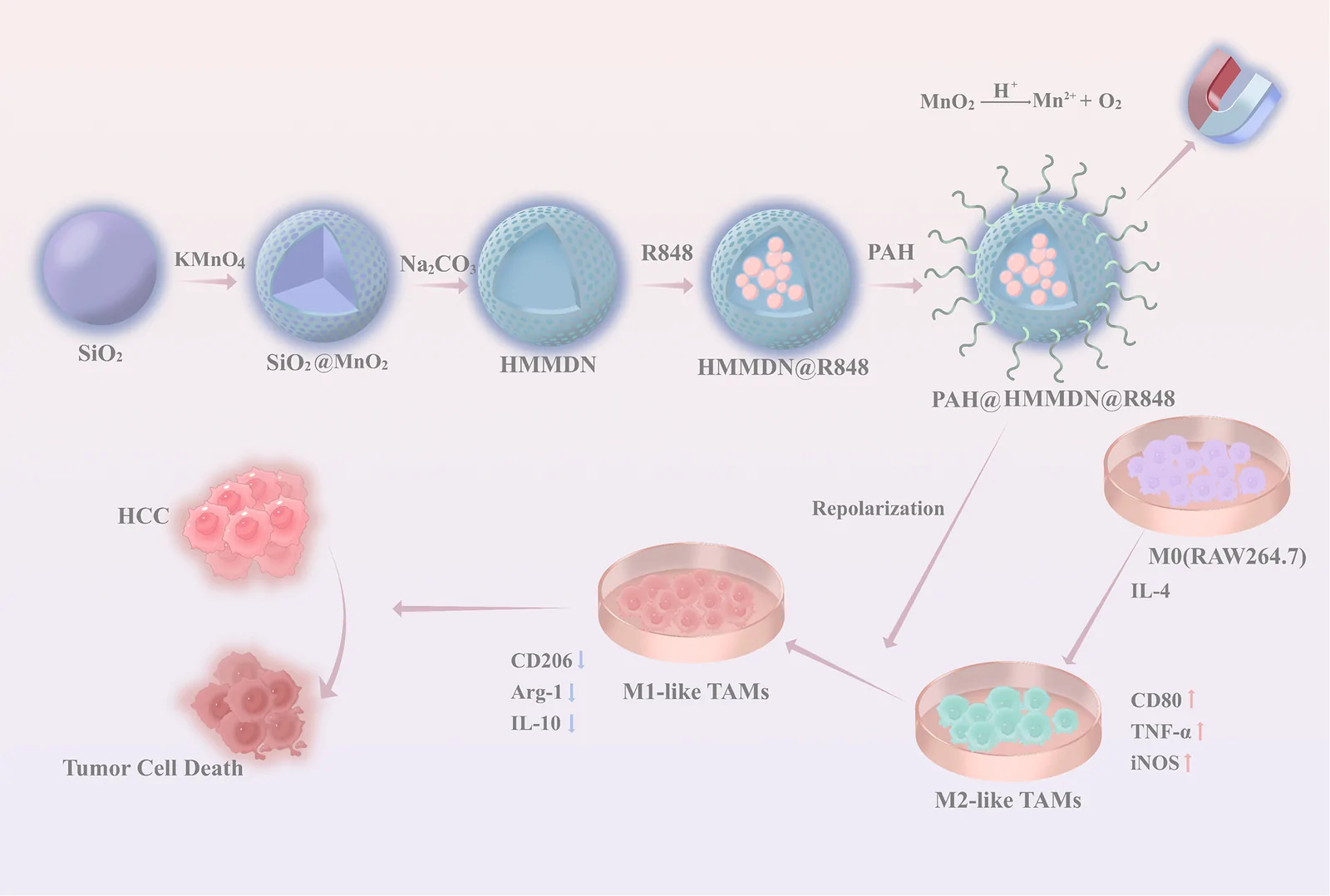
Schematic diagram showing the preparation of PAH@HMMDN@R848 and the *in vitro* inhibition of liver cancer cell growth by reprogramming M2-type macrophages. PAH, polyallylamine hydrochloride; HMMDN, Hollow manganese dioxide nanoparticles.

Aliquots (1.250 μL) of HMMDN were centrifuged (16,000 × g, 1 min), and the resulting precipitate was redispersed in 0.5 mL of DMF. Concurrently, 4 mg of R848 was weighed and dissolved in 1.5 mL of DMF. Both solutions were then combined in a 10 mL centrifuge tube and homogenized *via* ultrasonication for 45 min using the ultrasonic cell disruptor. In a separate step, 5 mg of PAH was dissolved in 1 mL of deionized water and vortex-mixed using a vortex mixer (SCI-VS, Scilogex, Rocky Hill, CT, USA) to ensure uniformity. This PAH solution was added to the previously sonicated mixture, and ultrasonic treatment was continued for an additional 45 min. Once the reaction was complete, the final mixture was centrifuged (16,000 × g, 15 min) and the precipitate was resuspended in 500 μL of deionized water, yielding a product that we designated as PAH@HMMDN@R848 (Fig 2).

#### 2.2.3. Measurement of Drug Loading Capacity and Encapsulation Efficiency.

In our study, the nanoparticle concentration was standardized based on the R848 content to ensure equivalent drug dosing across treatment groups. A calibration curve for R848 was constructed using DMF as the solvent. Solutions of R848 were prepared at varying concentrations (0, 87.5, 175, 350, 500, 750, 1000, 1500, 2000, 3000, 4000 and 6000 μg/mL). Following a 10-fold dilution, the ultraviolet absorption spectra of these solutions were recorded. Subsequently, the absorbance value at 291 nm for each R848 concentration was measured; these data points were then utilized to generate a standard curve.The PAH@HMMDN@R848 complex was then centrifuged to isolate a supernatant and a nanoparticle pellet. The absorbance of unencapsulated R848 present in the supernatant was quantified at 291 nm using ultraviolet-visible (UV-Vis) spectrophotometry using an ultraviolet-visible spectrophotometer (UV-26001, Shimadzu Corporation, Suzhou, China). The concentration of free R848 in the supernatant was determined by interpolation from the established calibration curve. Finally, the drug loading capacity (LC) and encapsulation efficiency (EE) were calculated using the following equations [1] and [2].


$$\begin{array}{c} LC(wt\%)=\frac{\text{mass of drug entrapped within the carriers}}{\text{total mass of drug}-\text{loaded carriers}}\times 100\% \end{array}$$ (1)

$$\begin{array}{c} EE(wt\%)=\frac{\text{mass of drug entrapped within the carriers}}{\text{total mass of drug initially added}}\times 100\% \end{array}$$ (2)

In this study, nanoparticle concentrations were standardized based on R848 content to ensure consistent dosing across treatment groups. Specifically, for experiments involving PAH@HMMDN@R848 and free R848, concentrations were normalized by R848 concentration, with in vivo administration at 10 mg/kg body weight and in vitro treatments expressed as equivalent R848 concentration (μg/mL). For MRI relaxivity assays, Mn²^+^ concentration (mM) was used as the benchmark, with samples diluted to identical Mn² ^+^ gradients (0.4, 0.2, 0.1, 0.05 mM). For cytotoxicity and hemolysis assays, the total mass concentration of PAH@HMMDN@R848 (μg/mL) was used, with gradients of 5, 10, 25, 50, and 100 μg/mL. When preparing treatment groups with equivalent R848 concentrations, the total nanoparticle mass required was calculated based on this loading to ensure consistent R848 levels between the free R848 and PAH@HMMDN@R848 groups.

### 2.3. Structural and functional analysis of nanoparticles

Morphology and dimensions of mesoporous SiO_2_, SiO_2_@MnO_2_, HMMDN, and PAH@HMMDN@R848 nanoparticles were investigated using a transmission electron microscope (TEM; HT7700, Hitachi High-Technologies Corporation, Tokyo, Japan). Hydrodynamic size and surface charge (zeta potential) were evaluated using a nanoparticle size and zeta potential analyzer (Zetasizer Zs90, Malvern Panalytical Ltd., Malvern, UK). In addition, the UV-Vis absorption profiles for R848, HMMDN, and PAH@HMMDN@R848 were recorded via UV-Vis spectroscopy using the ultraviolet-visible spectrophotometer. The colloidal stability of PAH@HMMDN@R848 was evaluated by monitoring hydrodynamic size changes in PBS containing 10% fetal bovine serum (FBS) at 37 °C over 72 h using the same DLS analyzer.

### 2.4. Evaluation of nanoparticle drug release profiles under *In Vitro* conditions

Next, we investigated the PAH@HMMDN@R848 release kinetics under four distinct conditions: PBS at pH 7.4, PBS at pH 6.5, PBS at pH 7.4 containing 1 mM GSH, and PBS at pH 6.5 containing 1 mM GSH. In each case, the PAH@HMMDN@R848 concentration was maintained at 100 μg/mL within a total volume of 1 mL. These sample mixtures were loaded into dialysis bags, which were subsequently suspended in centrifuge tubes filled with 3 mL of deionized water. The entire system was then subjected to constant agitation on an orbital shaker. At predetermined time intervals (0.5, 1, 2, 4, 8, 12, and 24 h), 100 μL aliquots were extracted from the solution surrounding the dialysis bags. Each aliquot was diluted to 1 mL with DMF. The concentration of released R848 was then determined by measuring the ultraviolet absorption peak at 291 nm using using the ultraviolet-visible spectrophotometer.

To determine the total R848 content encapsulated within PAH@HMMDN@R848, an identical concentration of the material was incubated in PBS 6.5 supplemented with 3 mM GSH. Following a 72-h dialysis procedure identical to that described above, the measured R848 concentration was considered to represent the total amount of the encapsulated drug. The cumulative drug release rate at each time point was then calculated as the ratio of the amount of R848 released relative to the total drug amount.

### 2.5. *In Vitro* evaluation of nanoparticle contrast efficacy for MRI

PAH@HMMDN@R848 was incubated separately with four distinct solutions: PBS at pH 7.4, PBS at pH 6.5, PBS at pH 7.4 supplemented with 1 mM GSH, and PBS at pH 6.5 supplemented with 1 mM GSH. In each incubation mixture, the Mn²^+^ concentration derived from PAH@HMMDN@R848 was set at 0.4 mM, with a total reaction volume of 2 mL. Following a 4-h incubation period, each of the four sample groups underwent serial dilution to achieve final Mn²^+^ concentrations of 0.4 mM, 0.2 mM, 0.1 mM, and 0.05 mM. The longitudinal relaxation times (T1) of the diluted samples were subsequently measured using a magnetic resonance analyzer (PQ001, Suzhou Niumag Analytical Instrument Corporation, Suzhou, China) operating at a magnetic field strength of 1 Tesla. For T1-weighted imaging, samples were scanned using a 3.0 T MRI scanner with a spin echo sequence (TR = 400–600 ms, TE = 10–20 ms, NEX = 1–2).

### 2.6. Cell culture

Mouse liver cancer (H22) cells were purchased from BDBIO Co., Ltd. (Zhejiang, China) while murine macrophage cells (RAW 264.7) and normal mouse hepatocytes (NCTC1469) were sourced from Procell Co., Ltd. (Wuhan, China). All cell lines were authenticated and tested negative for mycoplasma.H22 cells were maintained in RPMI 1640 medium supplemented with 10% (v/v) fetal bovine serum (FBS), 100 U/mL penicillin, and 0.1 mg/mL streptomycin. RAW 264.7 cells were grown in Dulbecco’s Modified Eagle Medium (DMEM) containing the same concentrations of FBS and antibiotics. NCTC1469 cells were cultured in NCTC 135 medium containing 10% horse serum and 2 mM glutamine. All cell lines were incubated in a humidified atmosphere at 37 °C with 5% CO_2_ in a CO_2_ incubator (STERI-CYCLE i160, Thermo Fisher Scientific, Waltham, MA, USA), and all manipulations were performed in a clean bench (1300SERIESA2, Thermo Fisher Scientific, Waltham, MA, USA). This study are commercially available, well-established cell lines, and that standard ethical guidelines were followed during their use.

### 2.7. Macrophage polarization

RAW 264.7 cells were plated in 6-well plates at a density of 1 × 10^4^ cells per well in complete medium and incubated for 24 h. Following this, the culture medium was removed. To induce M1 polarization, cells were stimulated with fresh medium containing 100 ng/mL lipopolysaccharide (LPS) and 25 ng/mL IFN-γ. Conversely, M2 polarization was induced by treating cells with fresh medium containing 25 ng/mL IL-4. These polarized macrophages were subsequently utilized for functional assays *in vitro*.

Macrophage phenotype was characterized with confocal laser scanning microscopy (CLSM) by evaluating the expression of an M1 surface marker (CD80) and an M2 surface marker (CD206) by immunostaining. Post-polarization, cells were fixed with 4% paraformaldehyde for 15 min, followed by permeabilization with 0.3% Triton X-100 for 30 min. After thorough washing with PBS, non-specific binding sites were blocked with 10% bovine serum albumin (BSA) solution for 1 h at room temperature. Cells were then incubated overnight at 4 °C with primary antibodies against CD80 (the dilution ratio is 1:100) and CD206. Following incubation with appropriate fluorescently labeled secondary antibodies for 2 h. Nuclei were counterstained using an anti-fade mounting medium containing 4’,6-diamidino-2-phenylindole (DAPI) (Sigma, D8417-1MG, Shanghai, China). Finally, samples were imaged and analyzed using a confocal laser scanning microscope (FV3000-RS, Olympus Corporation, Tokyo, Japan).

### 2.8. Hemolysis assay

Fresh blood from healthy SD rats was collected into anticoagulant tubes and centrifuged using the high-speed centrifuge at 1500 × g for 10 min to isolate red blood cells (RBCs). The RBCs were washed five times with sterile saline and then diluted to a 2% (v/v) suspension. Different concentrations of PAH@HMMDN@R848 (5, 10, 25, 50, and 100 μg/mL) were mixed with an equal volume of 2% RBC suspension and incubated at 37 °C for 3 h. Saline and 1% Triton X-100 were used as negative and positive controls, respectively. After incubation, the mixtures were centrifuged at 1000 × g for 5 min, and the absorbance of the supernatants at 540 nm was measured using a microplate reader. The hemolysis rate was calculated as: Hemolysis (%) = (ODsample – ODnegative)/ (ODpositive – ODnegative) × 100%.

### 2.9. Assessment of cytocompatibility and cytotoxicity

NCTC1469, H22, and RAW 264.7 macrophages were plated in 96-well plates at a density of 1 × 10^5^ cells per well. Following a 24-h incubation period, cells were incubated with the following treatment groups for 24 h: Group A (PBS control), Group B (free R848), Group C (HMMDN), and Group D (PAH@HMMDN@R848). Cell viability was assessed 24 h post-treatment using CCK-8 assays (Beyotime Biotechnology, Shanghai, China) with absorbance measured at 450 nm using the microplate reader. The proportion (%) of viable cells was determined using Equation [3].


$$\begin{array}{c} Cellviability(wt\%)=\frac{\text{ODsample }-\text{ ODblank}}{\text{ODcontrol }-\text{ ODblank}}\times \text{1}\text{0}\text{0}\% \end{array}$$ (3)

### 2.10. *In Vitro* Macrophage Repolarization Analysis by CLSM and Flow Cytometry

#### 2.10.1. CLSM.

Macrophage repolarization status was evaluated by detecting CD80 and CD206 surface marker expression using CLSM (IX51, Olympus, Japan). First, RAW 264.7 macrophages were seeded in 6-well plates (5 × 10^4^ cells/well) and cultured to adherence. Cells were polarized to the M2 phenotype following the established protocol. Subsequently, M2 macrophages were treated for 24 h with four experimental groups: Group A (PBS control), Group B (free R848), Group C (HMMDN), and Group D (PAH@HMMDN@R848). Immunofluorescence imaging was ultimately performed on all groups.

#### 2.10.2. Flow Cytometry.

For flow cytometry, RAW 264.7 cells were plated in 6-well plates (2 × 10^5^ cells/well) for overnight culture. After 12 h, M2 polarization was induced with IL-4 for 24 h. Polarized M2 macrophages were then exposed to the aforementioned treatment groups for 24 h, with untreated M2 macrophages serving as controls. Prior to analysis, adherent cells were trypsinized, pelleted by centrifugation (2,000 g, 5 min), and resuspended in 4% paraformaldehyde (PFA) for 10 min fixation. Following the removal of PFA, cells were blocked with 10% BSA for 30 min, permeabilized with 0.3% Triton X-100 (5 min, RT), stained with 5 µL FITC-conjugated anti-mouse CD80 or CD206 antibodies (4 °C, 1 h), washed, resuspended in 500 µL fixative solution, and analyzed for fluorescence intensity using a flow cytometer (SA3800, Sony Corporation, Tokyo, Japan).

### 2.11. *In Vitro* immune response activation

RAW 264.7 macrophages were plated in 24-well plates (5 × 10^4^ cells/well) for 24 h polarization to the M2 phenotype. These cells were then exposed to four experimental conditions for 24 h: Group A (PBS control), Group B (free R848), Group C (HMMDN), and Group D (PAH@HMMDN@R848). The levels of pro-inflammatory cytokines (IL-10, Arg-1, iNOS, TNF-α) in the supernatant were then quantified using ELISA kits (ELK Biotechnology, Wuhan, China). All assays included triplicate wells.

### 2.12. Assessment of anti-tumor activity

A co-culture system was established using 0.4 μm pore Transwell plates: M2 macrophages (5 × 10^4^ cells/well) in upper chambers and H22 hepatocarcinoma cells in lower chambers. Following 24 h of culture, macrophages were treated for 24 h with nanocomposites corresponding to Groups A-D as defined in section 2.10. Untreated M2/H22 co-cultures served as controls. H22 viability was measured by CCK-8 assays, while apoptosis was evaluated via flow cytometry according to manufacturer protocols.

### 2.13. Western blot analysis

Next we used western blotting to evaluate NF-κB p65 and IκBα phosphorylation in repolarized macrophages. RAW 264.7 cells were lysed in radio immunoprecipitation assay (RIPA) buffer (ASPEN Biotechnology., LTD, Wuhan, China) containing protease inhibitors (Protease Inhibitor Cocktail，04693159001，Roche，Shanghai，China). Rotein concentrations were determined by bicinchoninic acid assay (BCA) assay (ASPEN Biotechnology., LTD, Wuhan, China). Samples (40 μg/lane) were denatured in 5 × loading buffer (95 °C, 5 min), separated on 12.5% Bis-Tris gels (ASPEN Biotechnology., LTD, Wuhan, China) at 80V (30 min) then 100V (60 min), and transferred to polyvinylidene difluoride (PVDF) membranes. After blocking with 5% skimmed milk/TBS (1 h), membranes were probed with primary antibodies (4 °C, overnight) and horseradish peroxidase (HRP)-conjugated secondaries (RT, 30 min). Protein bands were visualized by enhanced chemiluminescence (ECL) detection (AS1059，ASPEN Biotechnology., LTD, Wuhan, China). The membrane was first incubated with a freshly prepared ECL solution applied dropwise. The resulting chemiluminescent signal was then visualized in the darkroom, with the exposure time being optimized for signal intensity before final development and fixation of the film. GAPDH served as the loading control.

### 2.14. Animals and tumor model

Male BALB/c nude mice (6 weeks old, 18–22 g) were purchased from Changzhou Cavens Laboratory Animal Co., Ltd. (Changzhou, China) and housed under specific pathogen-free conditions with a 12 h light/dark cycle and free access to food and water. All animal procedures were conducted in accordance with institutional and national guidelines for the care and use of laboratory animals (National Research Council, 2011) and reported in compliance with the ARRIVE guidelines (Kilkenny et al., 2014) [29,30]. The protocol was approved by the Institutional Animal Care and Use Committee of Suzhou Weiyuan Biotechnology Co., Ltd. (IACUC-20251215069). To minimize suffering, all mice were acclimatized for 7 days prior to the experiment, and all injections were performed under brief isoflurane anesthesia. Animals were monitored daily for signs of pain or distress, including changes in body weight, tumor appearance, mobility, feeding behavior, and grooming. Humane endpoints were predefined and strictly applied: mice were euthanized immediately if they exhibited any of the following criteria: (1) tumor diameter exceeding 1.5 cm in any direction or tumor volume exceeding 2,000 mm^3^; (2) body weight loss >20% of initial weight; (3) tumor ulceration, bleeding, or necrosis; (4) severe lethargy, persistent immobility, or inability to reach food or water; or (5) moribund condition as determined by the veterinarian. Any animal meeting these criteria was euthanized within 30 minutes of observation. No animals died before reaching these criteria during the study.To establish subcutaneous H22 tumors, 2 × 10⁶ H22 cells suspended in 100 μL PBS were injected into the right flank of each mouse. Tumor growth was monitored every other day. When tumors reached approximately 50–100 mm^3^ (about 5–7 days after inoculation), mice were randomly divided into four groups (n = 3 per group): (1) PBS control, (2) free R848 (10 mg/kg), (3) HMMDN (equivalent HMMDN dose as in group 4), and (4) PAH@HMMDN@R848 (R848 dose 10 mg/kg). Treatments were administered by intratumoral injection every 3 days for a total of 5 injections. Tumor dimensions were measured with calipers, and tumor volume was calculated using the formula V = (L × W^2^)/2, where L is the longest diameter and W is the shortest diameter. Body weight was recorded every 3 days. At day 15, mice were euthanized, tumors were excised, weighed, and photographed.

### 2.15. Histology and immunohistochemistry

Tumor tissues were fixed in 4% paraformaldehyde for 24 h, embedded in paraffin, and sectioned at 4 μm thickness using a microtome (SQ2125, Leica Biosystems, Wetzlar, Germany) and a spreading machine (PPTHK-21B, Leica Biosystems, Wetzlar, Germany). Hematoxylin and eosin (H&E) staining was performed for morphological evaluation. For immunohistochemistry (IHC), sections were deparaffinized, rehydrated, and subjected to antigen retrieval. After blocking with 10% goat serum, sections were incubated overnight at 4 °C with anti-CD80 or anti-CD206 primary antibodies. Then, sections were incubated with HRP-conjugated secondary antibodies, and color was developed using a DAB substrate kit. Nuclei were counterstained with hematoxylin. Images were captured using an inverted fluorescence microscope (CKX53, Olympus Corporation, Tokyo, Japan).

### 2.16. Cytokine Analysis in Tumor Tissues

Frozen tumor tissues were homogenized in ice-cold PBS (1:9, w/v) using an electric tissue homogenizer (PRO200, PRO Scientific Inc., Oxford, CT, USA). The homogenates were centrifuged at 12,000 × g for 15 min at 4 °C, and the supernatants were collected. Levels of TNF-α, iNOS, IL-10, and Arg-1 were measured using commercial ELISA kits according to the manufacturers' protocols. Absorbance was measured using the microplate reader. All samples were assayed in duplicate.

### 2.17. Statistical analysis

Data were analyzed with GraphPad Prism version 7.0 (GraphPad, San Diego, America). Normally distributed continuous variables are presented as mean ± standard deviation (SD) (x̄ ± s). Between-group comparisons were performed with unpaired t-tests (two groups) or analysis of variance (ANOVA; multiple groups). Significance thresholds were as follows: *P < 0.05, **P < 0.01, ***P < 0.001, ****P < 0.0001; ns = not significant.

### 2.18. Ethics statement

All animal experiments were approved by the Institutional Animal Care and Use Committee of Suzhou Weiyuan Biotechnology Co., Ltd. (IACUC-20251215069). All procedures were conducted in accordance with institutional and national guidelines for the care and use of laboratory animals (National Research Council, 2011) and reported in compliance with the ARRIVE guidelines (Kilkenny et al., 2014) [29,30]. Humane endpoints were predefined and strictly implemented as described in Section 2.14. All efforts were made to minimize animal suffering, including daily health monitoring, use of anesthesia during procedures, and immediate euthanasia upon reaching endpoint criteria

## 3. Results

### 3.1. Physicochemical characterization and stability of PAH@HMMDN@R848

**Fig 3:**
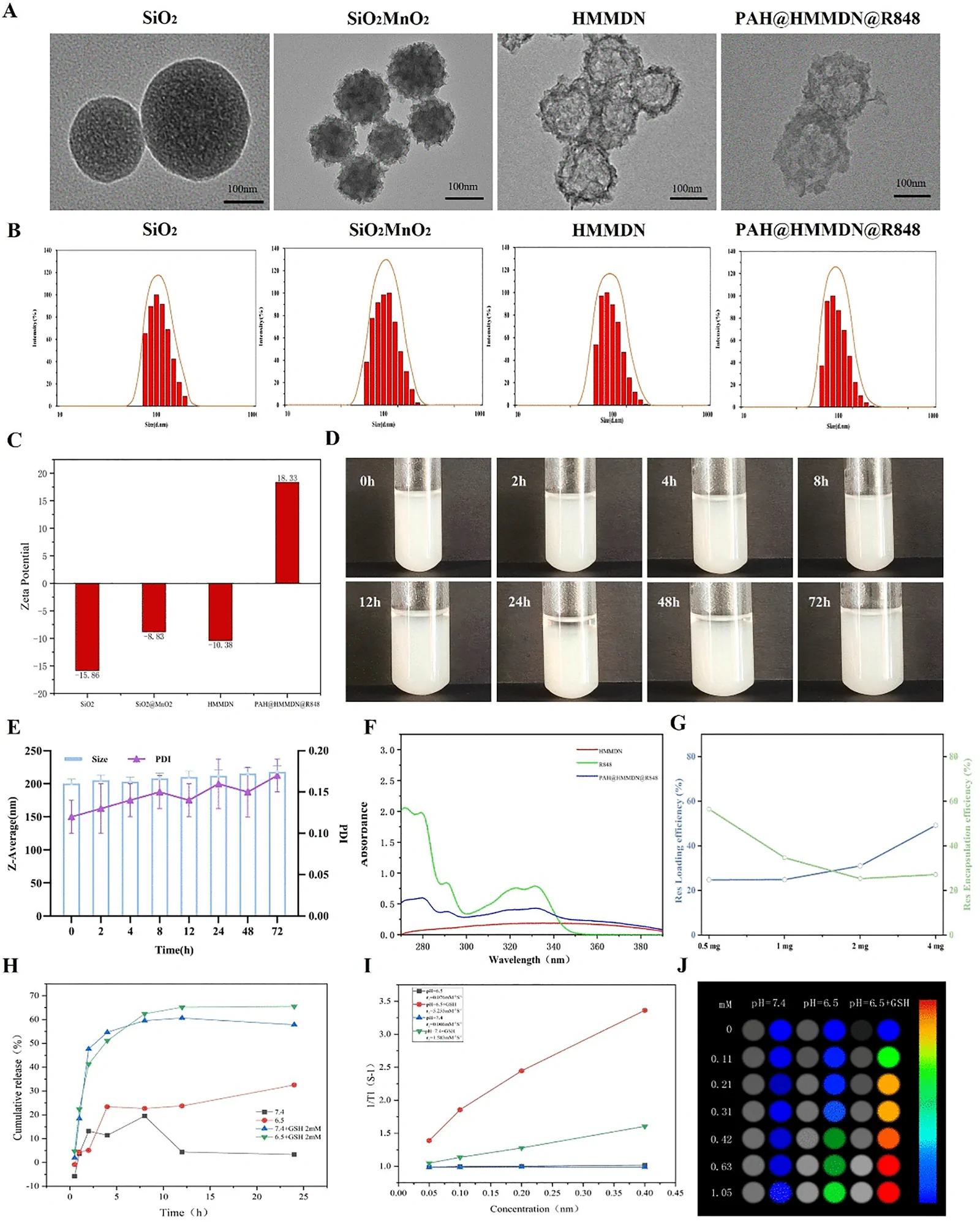
Characterization of PAH@HMMDN@R848. A. Transmission electron microscopy images of SiO_2_, SiO_2_@MnO_2_, HMMDN, and PAH@HMMDN@R848. Scale bar: 100 nm; B. Hydrodynamic diameters of SiO_2_, SiO₂@MnO_2_, HMMDN, and PAH@HMMDN@R848; C. Zeta potentials of SiO_2_, SiO_2_@MnO_2_, HMMDN, and PAH@HMMDN@R848; D-E. Size evolution of PAH@HMMDN@R848 in BSA over time. F. UV-vis absorption spectra of HMMDN, R848, and PAH@HMMDN@R848; G. Encapsulation efficiency and drug loading capacity of PAH@HMMDN@R848; H. Release profiles of PAH@HMMDN@R848 under different conditions; I. T1 relaxation curve of PAH@HMMDN@R848; J. T1-weighted body-of-water images of PAH@HMMDN@R848 at different manganese concentrations under various conditions, acquired using a 3.0T MRI scanner.

TEM images showed that SiO_2_ nanoparticles were uniformly spherical with a diameter of 96.4 ± 1.2 nm. After MnO_2_ coating and template removal, HMMDN exhibited a hollow structure with a diameter of 124.5 ± 0.5 nm. Following R848 loading and PAH coating, PAH@HMMDN@R848 maintained a uniform spherical morphology with a diameter of 163.9 ± 0.8 nm (Fig 3A). DLS analysis revealed an increase in hydrodynamic diameter from 125.93 ± 16.6 nm (HMMDN) to 167.1 ± 26.4 nm (PAH@HMMDN@R848), and zeta potential reversed from −10.38 ± 1.83 mV to +18.33 ± 4.8 mV, confirming successful R848 loading and PAH coating (Figs 3B, 3C). UV-vis spectroscopy showed that PAH@HMMDN@R848 retained the characteristic R848 absorption peak at 291 nm (Fig 3F), with a drug loading capacity of 49.20% and encapsulation efficiency of 27.13% (Fig 3G). Notably, the colloidal stability of PAH@HMMDN@R848 in PBS containing 10% FBS remained excellent over 72 h, with no significant aggregation (PDI < 0.3) (Figs 3D, 3E), addressing concerns about potential instability under physiological conditions.

### 3.2. TME-Responsive Drug Release and MRI Contrast Enhancement

The in *vitro* release profile of PAH@HMMDN@R848 exhibited clear pH/GSH dual responsiveness (Fig 3H). At pH 7.4, drug release was minimal (3.4% at 24 h), whereas at pH 6.5 (mimicking acidic TME), release increased to 32.51%. Addition of 1 mM GSH dramatically accelerated release, reaching 57.82% at pH 7.4 + GSH and 65.55% at pH 6.5 + GSH. This enhanced release under acidic and reducing conditions is attributed to the degradation of MnO_2_ into Mn^2+^ , which disrupts the nanoparticle structure [27]. Such TME-responsive behavior is crucial for minimizing systemic toxicity while maximizing intratumoral drug delivery.

Consistent with drug release, the MRI contrast performance of PAH@HMMDN@R848 was also pH/GSH dependent. The longitudinal relaxivity (r_1_) at pH 7.4 was negligible (0.006 mM ^-^^1^s ^-^^1^), but increased 12.7-fold at pH 6.5 (0.076 mM ^-1^s ^-1^) (Fig 3I). In the presence of 10 mM GSH, r₁ rose dramatically to 1.583 mM ^-^^1^s ^-^^1^ (pH 7.4) and 5.255 mM ^-^^1^s ^-^^1^ (pH 6.5), surpassing the clinical contrast agent Gd-DTPA (4.49 mM ^-^^1^s ^-^^1^) [31]. T1-weighted phantom images confirmed the brightest signals under combined acidic and reducing conditions (Fig 3J). These results demonstrate that Mn^2+^ released from HMMDN acts as an efficient T_1_ contrast agent only in the TME, enabling "smart" imaging with minimal background signal, a feature highly desirable for tumor-specific diagnosis.

### 3.3. Biocompatibility Assessment

**Fig 4:**
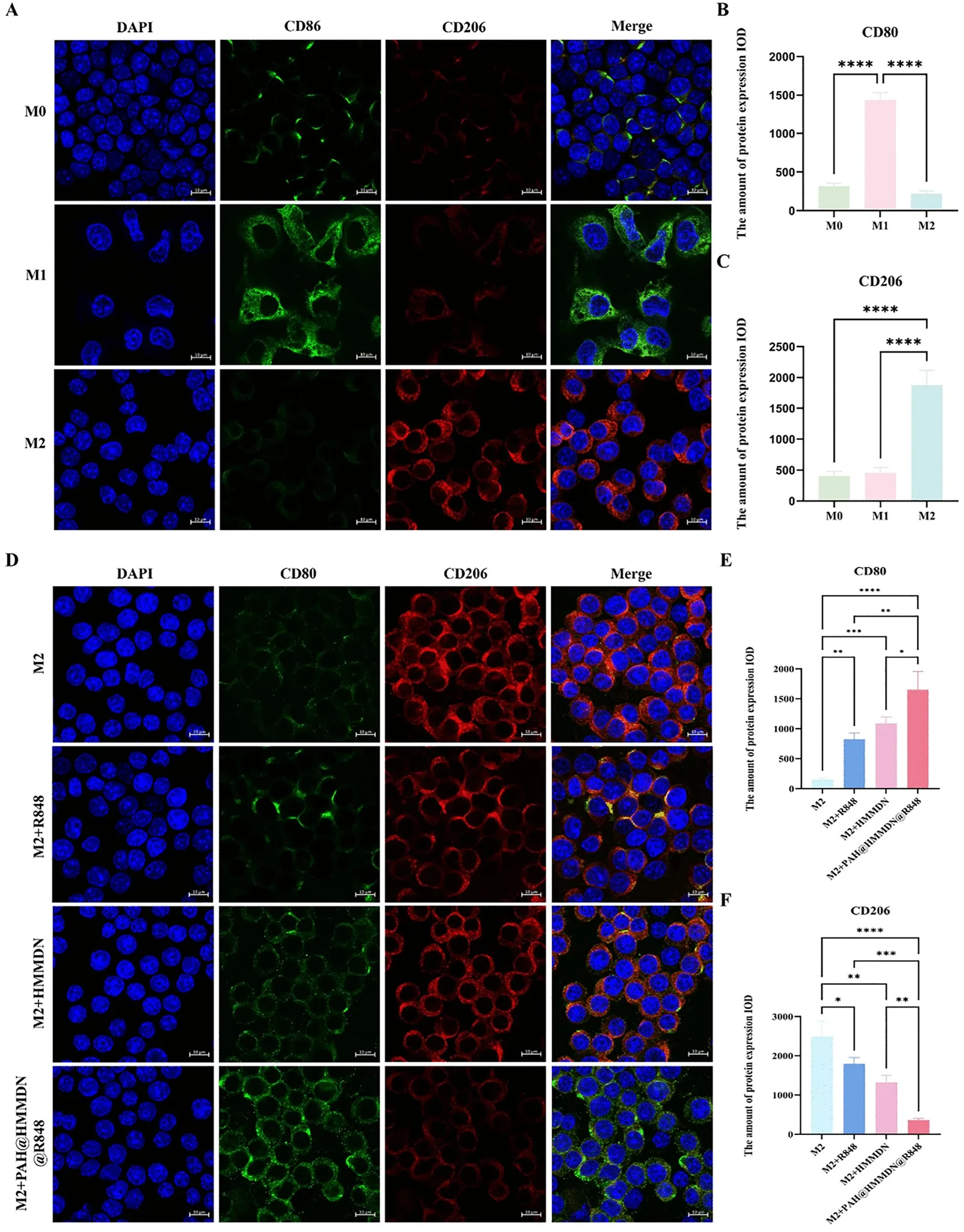
Expression of CD80 and CD206 in Different Macrophage States. A. Confocal laser scanning microscopy results (scale bar: 10 µm); B. Quantitative analysis of CD80 fluorescence intensity in different macrophage states; C. Quantitative analysis of CD206 fluorescence intensity in different macrophage states; D. In vitro fluorescence images of CD80 and CD206 expression in M2 macrophages treated with R848, HMMDN, and PAH@HMMDN@R848 (scale bar: 10 µm); E. Quantitative analysis of CD80 fluorescence intensity on M2 macrophages treated with different nanoparticles; F. Quantitative analysis of CD206 fluorescence intensity on M2 macrophages treated with different nanoparticles. PAH, polyallylamine hydrochloride; HMMDN, hollow manganese dioxide nanoparticles; CLSM, confocal laser scanning microscope. n = 3。*P < 0.05, **P < 0.01, ***P < 0.001, ****P < 0.0001.

Biocompatibility is a prerequisite for in vivo application. The hemolysis assay showed that PAH@HMMDN@R848 caused negligible hemolysis (<5%) even at 100 μg/mL (Figs S1A in S1 File, Fig 4B), confirming excellent blood compatibility. CCK-8 assays revealed that the nanoplatform was non-toxic to normal hepatocytes (NCTC1469) and RAW264.7 macrophages at concentrations up to 25 μg/mL, but exhibited selective cytotoxicity against H22 tumor cells at 50 μg/mL (Fig S1C in S1 File). This differential toxicity likely stems from the higher metabolic activity and glutathione levels in cancer cells, which accelerate MnO₂ degradation and subsequent Mn^2+^ release. The favorable safety profile supports further *in vivo* evaluation.

### 3.4. *In Vitro* Repolarization of M2 Macrophages by PAH@HMMDN@R848

**Fig 5:**
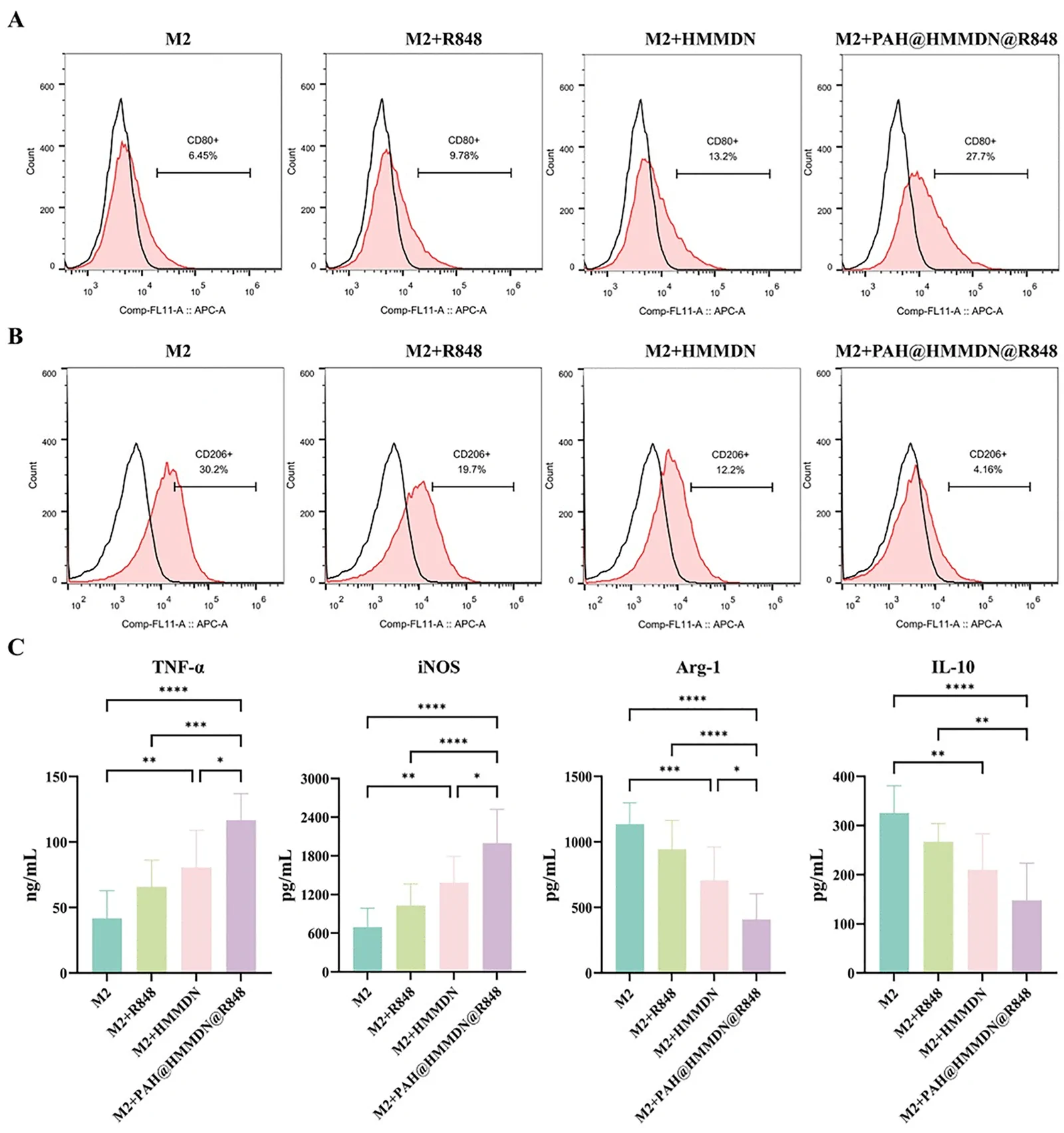
Validation of macrophage phenotypic reprogramming. A. Flow cytometric analysis of CD80 expression in M2 macrophages treated with different nanoparticles in vitro; B. Flow cytometric analysis of CD206 expression in M2 macrophages treated with different nanoparticles in vitro; C. Levels of immune cytokines (including TNF-α, iNOS, Arg-1, and IL-10) in the culture supernatants of M2 macrophages from different treatment groups. n = 3。*P < 0.05, **P < 0.01, ***P < 0.001, ****P < 0.0001.

Macrophage polarization was first validated by treating RAW264.7 cells with LPS/IFN-γ (M1 inducer) or IL-4 (M2 inducer). CLSM confirmed that LPS/IFN-γ significantly upregulated the M1 marker CD80, while IL-4 increased the M2 marker CD206 (Figs 4A–4C). When M2-polarized macrophages were treated with PAH@HMMDN@R848, CD80 fluorescence intensity increased most strongly, while CD206 fluorescence decreased most markedly among all groups (Figs 4D–4F). Flow cytometry quantification revealed that PAH@HMMDN@R848 increased CD80 ^+^ cells to 27.7% and reduced CD206 ^+^ cells to 4.16%, significantly outperforming free R848 (13.2% CD80 ^+^ , 19.8% CD206 ^+^) and HMMDN alone (9.78% CD80 ^+^ , 52.3% CD206 ^+^) (Figs 5A, 5B). ELISA further showed that PAH@HMMDN@R848 significantly elevated M1-related cytokines (iNOS, TNF-α) and suppressed M2-related cytokines (Arg-1, IL-10) (Fig 5C). Complementary flow cytometry and western blotting analyses yielded consistent results (Figs 5A-B and Figs S2A-B in S1 File). These data demonstrate that the nanoplatform effectively reprograms M2 macrophages toward the M1 phenotype, with efficacy superior to either component alone, suggesting a synergistic effect.

### 3.5. Synergistic Activation of NF-κB and STING Pathways Underlies Repolarization

To uncover the molecular mechanism, we examined the activation of NF-κB and STING pathways by Western blot. PAH@HMMDN@R848 strongly enhanced phosphorylation of p65 and IκBα compared to R848 or HMMDN alone (Figs S3A, S3C in S1 File), indicating robust NF-κB activation, consistent with R848's known TLR7/8 agonism [19,20]. Importantly, PAH@HMMDN@R848 also increased phosphorylation of TBK1 and IRF3, key STING pathway components, to a greater extent than HMMDN alone (Figs S3B, S3D in S1 File). This confirms that Mn^2+^ released from HMMDN activates the cGAS-STING pathway, as reported previously [26,32]. The concurrent activation of both pathways provides a mechanistic basis for the observed synergistic repolarization: R848 triggers TLR-NF-κB signaling, while Mn^2+^ engages STING-TBK1-IRF3 signaling, and crosstalk between these pathways amplifies the pro-inflammatory M1 response [33,34]. This dual activation strategy represents a powerful approach to TAMs reprogramming.

### 3.6. PAH@HMMDN@R848 Enhances Macrophage-Mediated Tumor Cell Killing *In Vitro*

**Fig 6:**
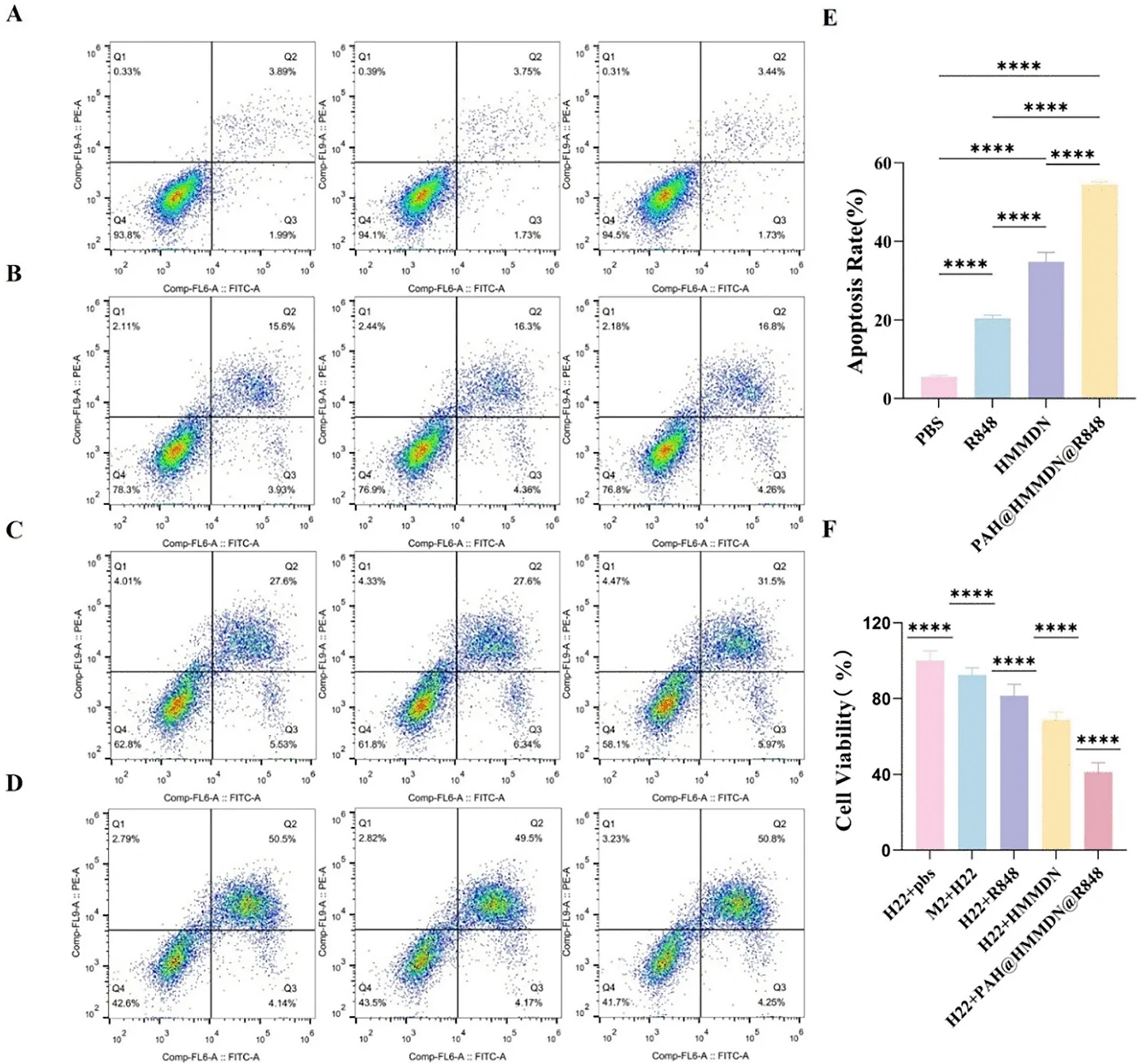
Apoptosis assays and in vitro antitumor activity evaluation. A–E. Survival rates of H22 cells treated with PBS, R848, HMMDN, and PAH@HMMDN@R848; F. Lysis of H22 cells after co-incubation with M2-type tumor-associated macrophages treated with different nanoparticles in a Transwell system. n = 3. ****P < 0.0001.

In a Transwell co-culture system modeling macrophage-tumor cell interaction, PAH@HMMDN@R848 treatment of M2 macrophages led to the highest apoptosis rate in H22 tumor cells (54.45%), significantly exceeding free R848 (20.42%) and HMMDN alone (34.85%) (Figs 6A–6E). Correspondingly, H22 cell viability was lowest in the PAH@HMMDN@R848 group (41.2%) compared to R848 (81.6%) and HMMDN (68.8%) (Figs 6F). Since the nanoplatform itself showed no direct cytotoxicity at the concentration used (25 μg/mL, Figs S1C in S1 File), the observed anti-tumor effect is attributable to macrophage repolarization. These results indicate that reprogrammed M1 macrophages acquire potent tumoricidal activity, likely through secretion of pro-inflammatory cytokines and enhanced phagocytosis [35].

### 3.7. *In Vivo* Anti-Tumor Efficacy and TME Remodeling

**Fig 7:**
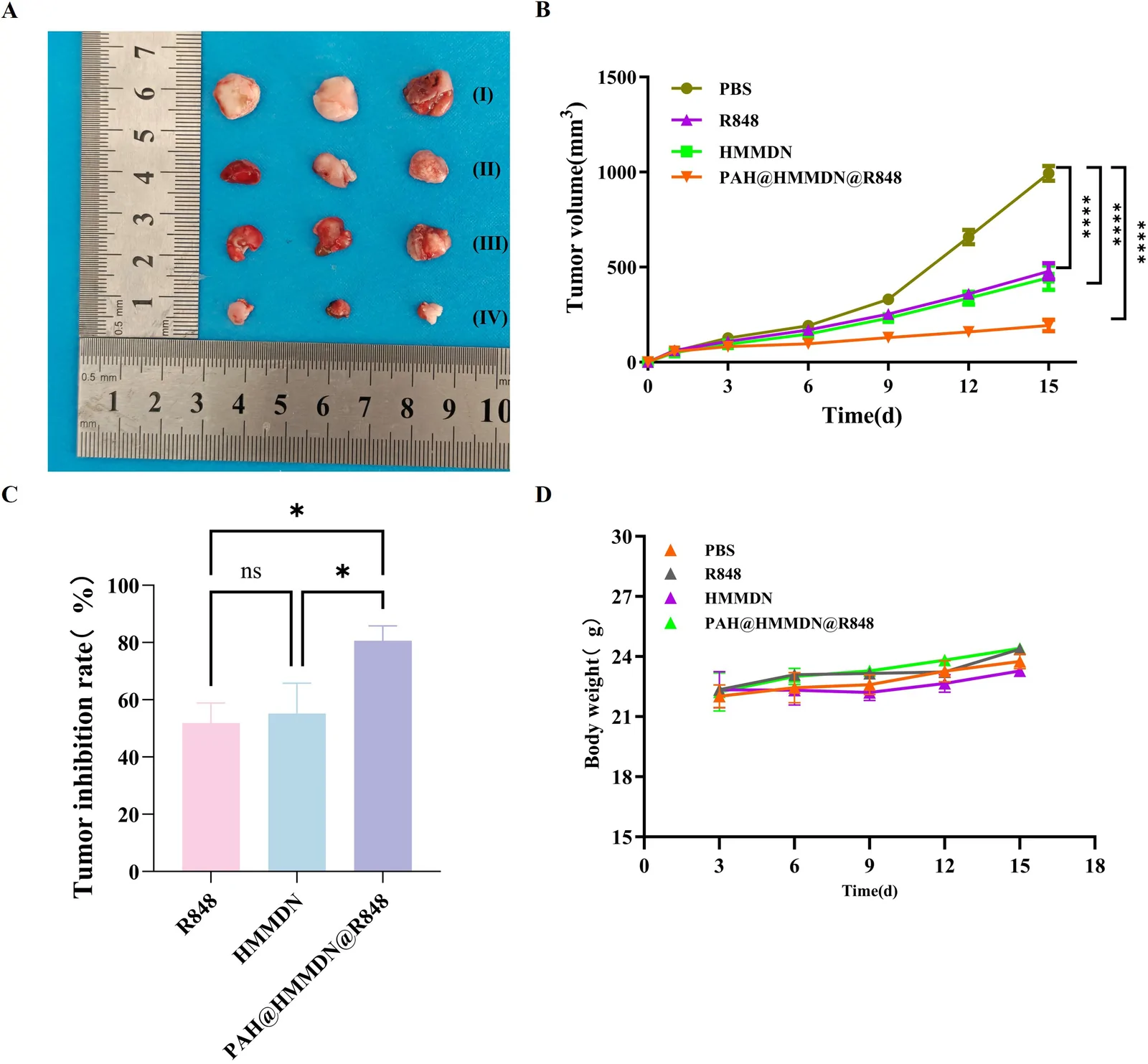
In vivo experimental results. A. Comparison of tumor growth after 16 days of different treatments. (I) PBS control group; (II) R848 group; (III) HMMDN group; (IV) PAH@HMMDN@R848 group; B. Body weight measurements of mice in each group; C. Tumor inhibition rates in each group on day 15 post-treatment; D. Tumor growth curves of mice in different treatment groups. n = 3. *P < 0.05, **P < 0.01, ***P < 0.001, ****P < 0.0001, ns indicates no statistical significance.

In a subcutaneous H22 tumor-bearing BALB/c mouse model, the *in vivo* anti-tumor efficacy and safety of PAH@HMMDN@R848 were further evaluated via intratumoral injection. During the 15-day treatment course, the body weights of mice in all treatment groups maintained a steady increase (Fig 7B), showing no significant difference compared with the PBS control group. However, the tumor growth curves (Fig 7D), final tumor photographs (Fig 7A), and tumor volume and weight statistics (Fig 7C) exhibited significant differences. The results showed that tumors in the PBS control group grew rapidly, with a final average volume of 992.7 ± 68.1 mm^3^ and an average weight of 0.415 ± 0.0085 g. Both R848 monotherapy and HMMDN monotherapy demonstrated certain tumor inhibition effects, with tumor volumes and weights significantly lower than those in the control group (*P* < 0.01), achieving tumor inhibition rates of 51.9% and 55.3%, respectively. However, in the PAH@HMMDN@R848 combination treatment group, the final average tumor volume was only 192.9 ± 52.6 mm^3^, the average weight was as low as 0.042 ± 0.0104 g, and the tumor inhibition rate reached as high as 80.6%, with its efficacy being significantly superior to either single-component treatment group (*P* < 0.05).

**Fig 8:**
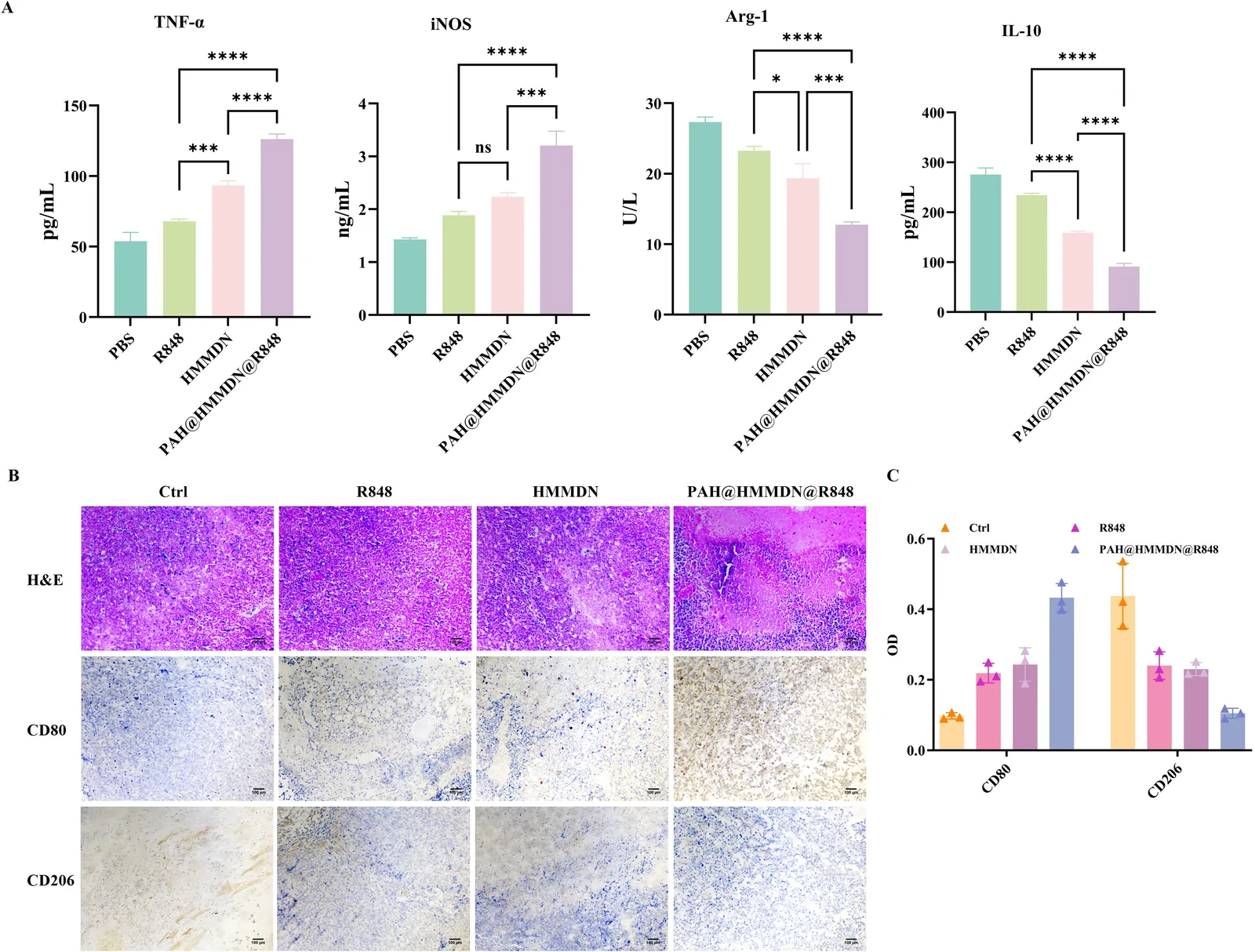
Immunocytochemical, H&E, and Immunohistochemical Staining. A. Serum levels of TNF-α, iNOS, Arg-1, and IL-10 in mice from each group; B, C. H&E staining and CD80, CD206 immunohistochemical staining of tumor tissues from different groups (scale bar: 100μm). n = 3. *P < 0.05, **P < 0.01, ***P < 0.001, ****P < 0.0001; ns indicates no statistical significance.

The immunological mechanism underlying the *in vivo* efficacy was investigated by detecting the levels of key cytokines in tumor tissue homogenates (Fig 8A). The results showed that the expression levels of pro-tumor M2-type related factors Arg-1 and IL-10 were significantly inhibited, decreasing by approximately 53% and 67%, respectively; whereas the expression of anti-tumor M1-type related factors iNOS and TNF-α was strongly induced, increasing by approximately 125% and 134%, respectively, which echoed the *in vitro* experimental results.

IHC staining was performed on tumor tissues (Figs 8B-C). The staining results for CD80 (M1 marker) and CD206 (M2 marker) provided direct evidence of spatial distribution. The results showed that in tumors of the PBS control group, scattered CD206-positive areas (brown) were visible, while CD80-positive signals were scarce. In the R848 or HMMDN treatment groups, CD80-positive areas increased, and CD206-positive areas correspondingly decreased. However, in tumor sections from the PAH@HMMDN@R848 treatment group, extensive and dense infiltration of CD80-positive cells was observed, while CD206-positive signals became extremely weak.

## 4. Discussion

In this study, we successfully constructed a multifunctional nanoplatform, PAH@HMMDN@R848, that integrates TME-responsive drug release, MRI contrast, and synergistic activation of NF-κB and STING pathways for effective TAMs reprogramming in liver cancer. The platform exhibited uniform spherical morphology (~164 nm), favorable colloidal stability, and pH/GSH dual-responsive drug release, achieving 65.55% cumulative R848 release within 24 h under simulated TME conditions (pH 6.5 + GSH). The released Mn^2+^ not only served as an efficient T₁ MRI contrast agent (r_1_ = 5.255 mM ^- 1^s ^- 1^ at pH 6.5 + GSH) but also activated the STING pathway, complementing R848-induced NF-κB activation to synergistically drive M2-to-M1 macrophage repolarization. Both in vitro and in vivo studies demonstrated that PAH@HMMDN@R848 potently repolarized M2 macrophages, reshaped the immunosuppressive TME, and significantly inhibited tumor growth in a subcutaneous H22 model (80.6% inhibition) without overt toxicity.

The physicochemical properties of PAH@HMMDN@R848 are critical for its biological performance. The nanoparticle size (~164 nm) falls within the optimal range for passive tumor targeting via the enhanced permeability and retention (EPR) effect, as tumor vascular endothelial gaps typically range from 100 to 800 nm [4]. The positive surface charge (+18.33 mV) after PAH coating not only confirmed successful modification but also may enhance cellular uptake by macrophages. Importantly, the nanoplatform maintained excellent colloidal stability in serum-containing medium over 72 h (PDI < 0.3), addressing a key concern raised during peer review regarding potential aggregation under physiological conditions. This stability is likely due to the hydrophilic nature of PAH and its electrostatic interactions with the nanoparticle core, preventing protein adsorption and subsequent aggregation [22].

The pH/GSH dual-responsive drug release observed in this study is a direct consequence of HMMDN's TME-triggered degradation. MnO_2_ is known to decompose under acidic conditions (e.g., pH 6.5 in tumors) and in the presence of high glutathione concentrations (typically 2–10 mM in cancer cells vs. 2–20 μM in normal tissues) [36]. Our data showed minimal release at pH 7.4 without GSH (3.4% at 24 h), but dramatically accelerated release under combined acidic and reducing conditions (65.55% at 24 h), consistent with previous reports on MnO_2_-based nanocarriers [28,36]. This TME-responsive behavior minimizes premature drug leakage during circulation while ensuring high local drug concentration at the tumor site, a key advantage for reducing systemic toxicity and enhancing therapeutic efficacy.

The released Mn^2+^ ions from HMMDN degradation served a dual role: as a T₁ MRI contrast agent and as an immunomodulator. The r_1_ relaxivity reached 5.255 mM ^-^^1^s ^- 1^ under pH 6.5 + GSH, surpassing the clinical contrast agent Gd-DTPA (4.49 mM ^- 1^s ^- ^- 1^^) [37]. This value is comparable to other manganese-based contrast agents [31], but with the added advantage of TME-specific activation, enabling "smart" imaging with minimal background signal. Such features are highly desirable for tumor-specific diagnosis and real-time monitoring of treatment response.

Biocompatibility is a prerequisite for clinical translation. Our nanoplatform showed excellent blood compatibility (hemolysis <5%) and selective cytotoxicity against H22 tumor cells at higher concentrations (50 μg/mL) while remaining safe for normal hepatocytes and macrophages at concentrations up to 25 μg/mL. This selective toxicity likely reflects the higher metabolic activity and glutathione levels in cancer cells, which accelerate MnO_2_ degradation and subsequent Mn^2+^ release. The absence of direct cytotoxicity at the concentration used for co-culture experiments (25 μg/mL) confirms that the observed anti-tumor effects are primarily mediated by macrophage repolarization rather than direct nanoparticle toxicity.

The core finding of this study is that PAH@HMMDN@R848 achieves synergistic activation of the NF-κB and STING pathways. Regarding the NF-κB pathway, compared with the PBS control group, free R848 treatment induced a certain degree of p65 and IκBα phosphorylation, which is consistent with its known mechanism as a TLR7/8 agonist [38]. HMMDN treatment alone also induced slight NF-κB pathway activation, which may originate from stimulation by its degradation product Mn^2+^ or other physicochemical properties. However, PAH@HMMDN@R848 treatment elicited the strongest p-p65 and p-IκBα signals, with gray value ratios of the protein bands significantly higher than those in any single treatment group, indicating that the nanocomplex can synergistically and greatly enhance NF-κB pathway activation. Regarding the STING pathway, p-TBK1 and p-IRF3 levels were elevated in the HMMDN treatment group, confirming that Mn^2+^ can act as a cofactor for cGAS or directly activate STING. The PAH@HMMDN@R848 treatment group not only inherited this effect but also elevated the phosphorylation levels of TBK1 and IRF3 to an even higher degree.

The in vivo efficacy of PAH@HMMDN@R848 was demonstrated in a subcutaneous H22 tumor model. Intratumoral injection of the nanoplatform achieved 80.6% tumor inhibition, significantly higher than free R848 (51.9%) or HMMDN alone (55.3%). The superior efficacy can be attributed to several factors: (1) TME-responsive release ensures sustained local drug concentration; (2) PAH coating enhances R848 solubility and cellular uptake; (3) synergistic activation of NF-κB and STING pathways maximizes M1 repolarization; and (4) Mn^2+^ -mediated STING activation further enhances the immunostimulatory environment. Immunohistochemistry and cytokine analysis of tumor tissues confirmed that PAH@HMMDN@R848 treatment increased M1 macrophages (CD80^+^) and M1 cytokines (TNF-α, iNOS), while decreasing M2 macrophages (CD206⁺) and M2 cytokines (IL-10, Arg-1). These results demonstrate that the nanoplatform effectively remodels the immunosuppressive TME, converting "cold" tumors into "hot" tumors susceptible to immune attack.

Despite these promising results, several limitations should be acknowledged. First, the in vivo evaluation was performed using a subcutaneous tumor model and intratumoral injection, which may not fully recapitulate the clinical scenario of orthotopic liver cancer with systemic delivery. Subcutaneous models lack liver-specific microenvironmental components such as sinusoidal endothelial cells and Kupffer cells, and their vascularization and immune infiltration patterns differ from orthotopic tumors [39]. Future studies should explore intravenous administration in orthotopic models and assess pharmacokinetics, biodistribution, and long-term toxicity. Second, while we demonstrated STING pathway activation, we did not perform inhibition studies (e.g., using STING or NF-κB inhibitors) to confirm causality; such experiments would further strengthen the mechanistic conclusions. Third, the current platform relies on passive targeting via the EPR effect. Active targeting strategies, such as conjugation with ligands specific to TAMs (e.g., anti-CD206 antibodies) or tumor cells, could enhance tumor accumulation and selectivity. Fourth, the study duration (15 days) was relatively short; longer-term observation is needed to assess potential chronic toxicity and manganese accumulation. Finally, combining this nanoplatform with immune checkpoint inhibitors (e.g., anti-PD-1) may produce even greater therapeutic efficacy, as suggested by recent studies [40].

## 5. Conclusion

In summary, we successfully engineered a multi-functional nanoplatform PAH@HMMDN@R848. In vitro and in vivo investigations demonstrated its dual capabilities: excellent MRI contrast properties and TME-responsive targeting. The therapeutic mechanism involves synergistic activation of NF-κB and STING pathways, driving M2-to-M1 macrophage repolarization and converting the immunosuppressive tumor milieu into an immunostimulatory state, ultimately suppressing liver cancer growth. These findings highlight the translational prospects of PAH@HMMDN@R848 for liver cancer theranostics and provide a foundation for further development.

## Supporting information

S1 File Supporting figures and tables. This file contains Figures S1–S3.
(DOCX)

S1 Dataset Raw numerical data underlying all figures and statistical analyses. (ZIP)

S1 Checklist Completed PLOS ONE Humane Endpoints Checklist. (DOCX)

S1 Raw images Original uncropped and unadjusted electrophoresis images. (PDF)
