# Supplementary material for: Reprogramming M2 macrophages via TLR7/8 agonist-loaded hollow MnO2 nanovehicles to suppress the progression of liver cancer
Source: PLoS One. 2026 Sep 18;21(9):e0358567. doi: 10.1371/journal.pone.0358567 (PMC13588417; doi:10.1371/journal.pone.0358567)
Supplement: Supplementary file 1 [file pone.0358567.s004.pdf]

Arg-1（FigS2A）

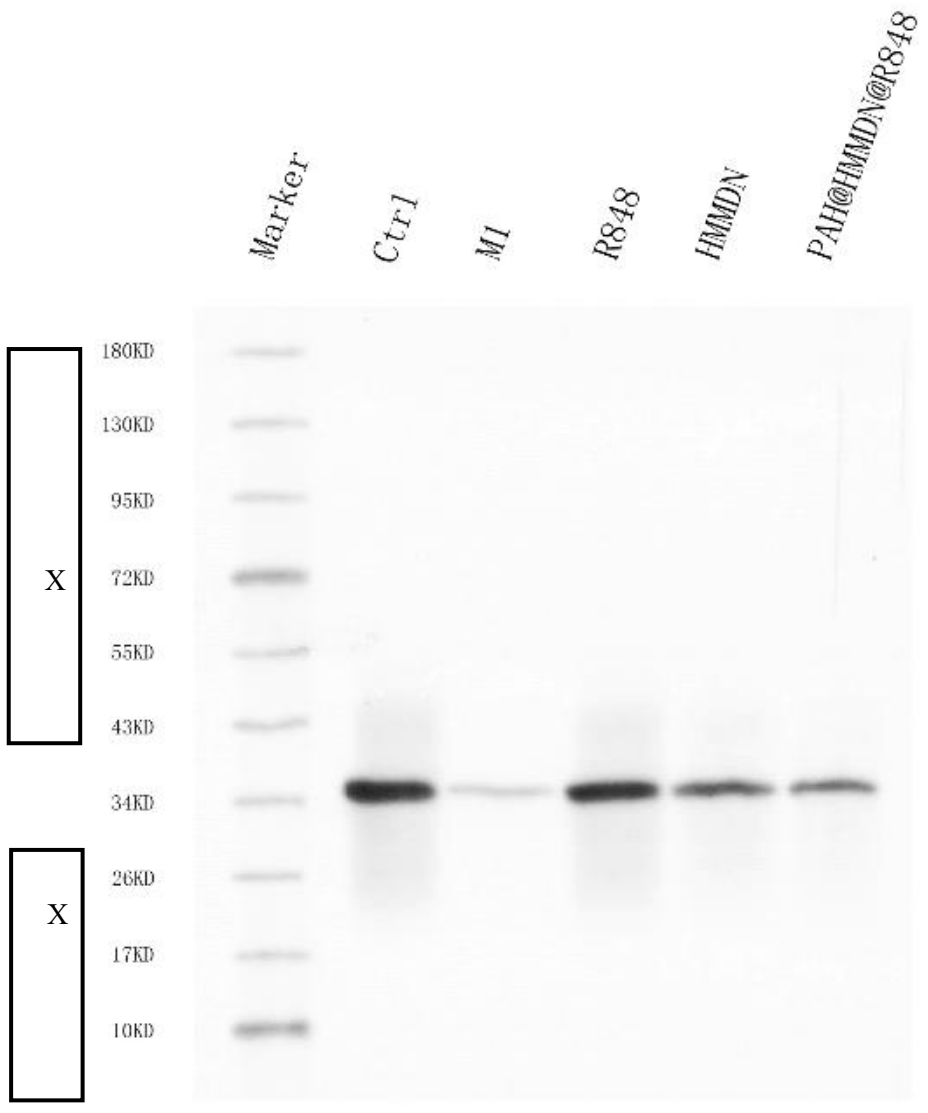

Marker
Ctrl
M1
R848
HMMDN
PAH@HMMDN@R848
180KD
130KD
95KD
72KD
55KD
43KD
34KD
26KD
17KD
10KD
X
X

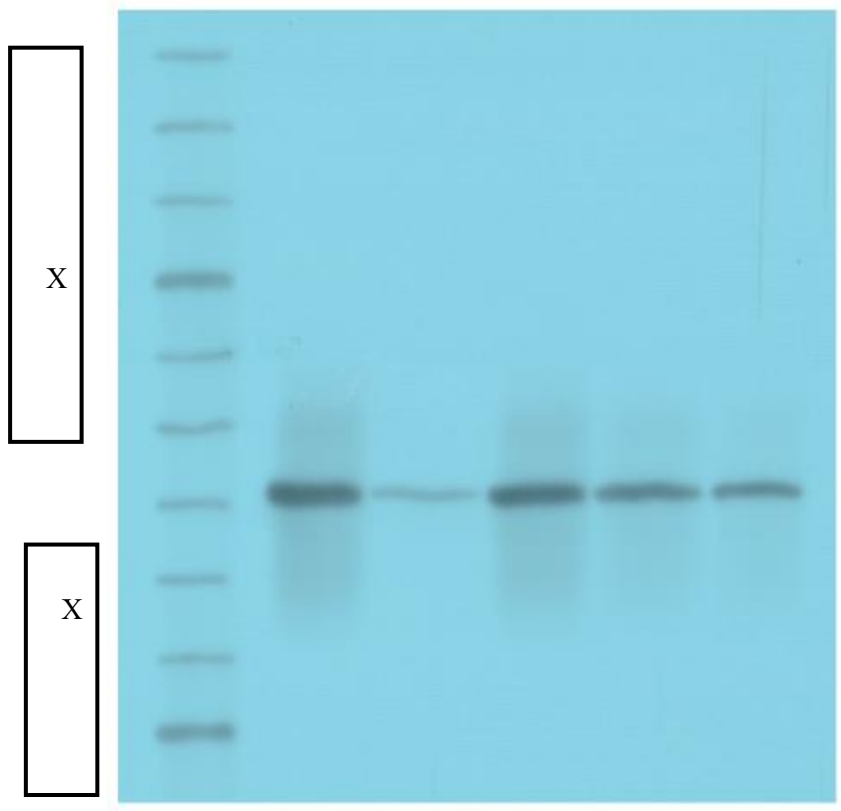

X
X

CD80（FigS2A）

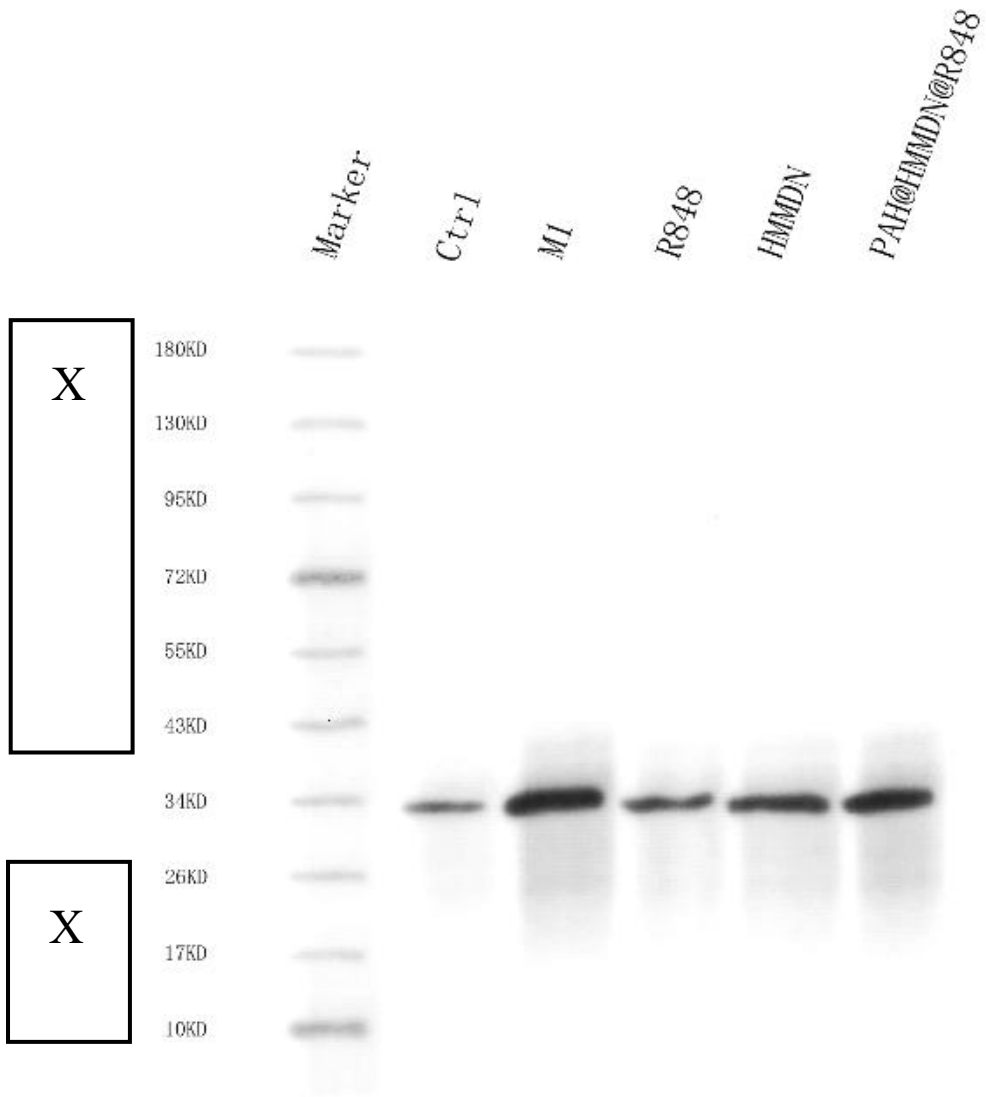
Marker
Ctrl
M1
R848
HMMDN
PAH@HMMDN@R848
X
X
180KD
130KD
95KD
72KD
55KD
43KD
34KD
26KD
17KD
10KD

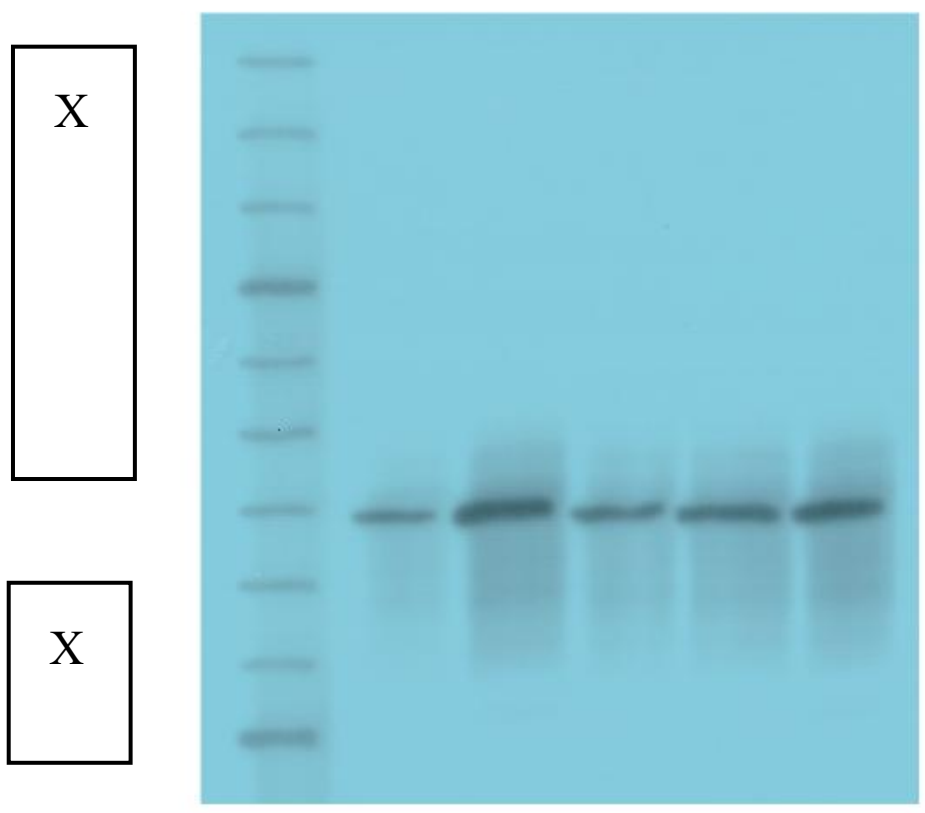
X
X

CD206（FigS2A）

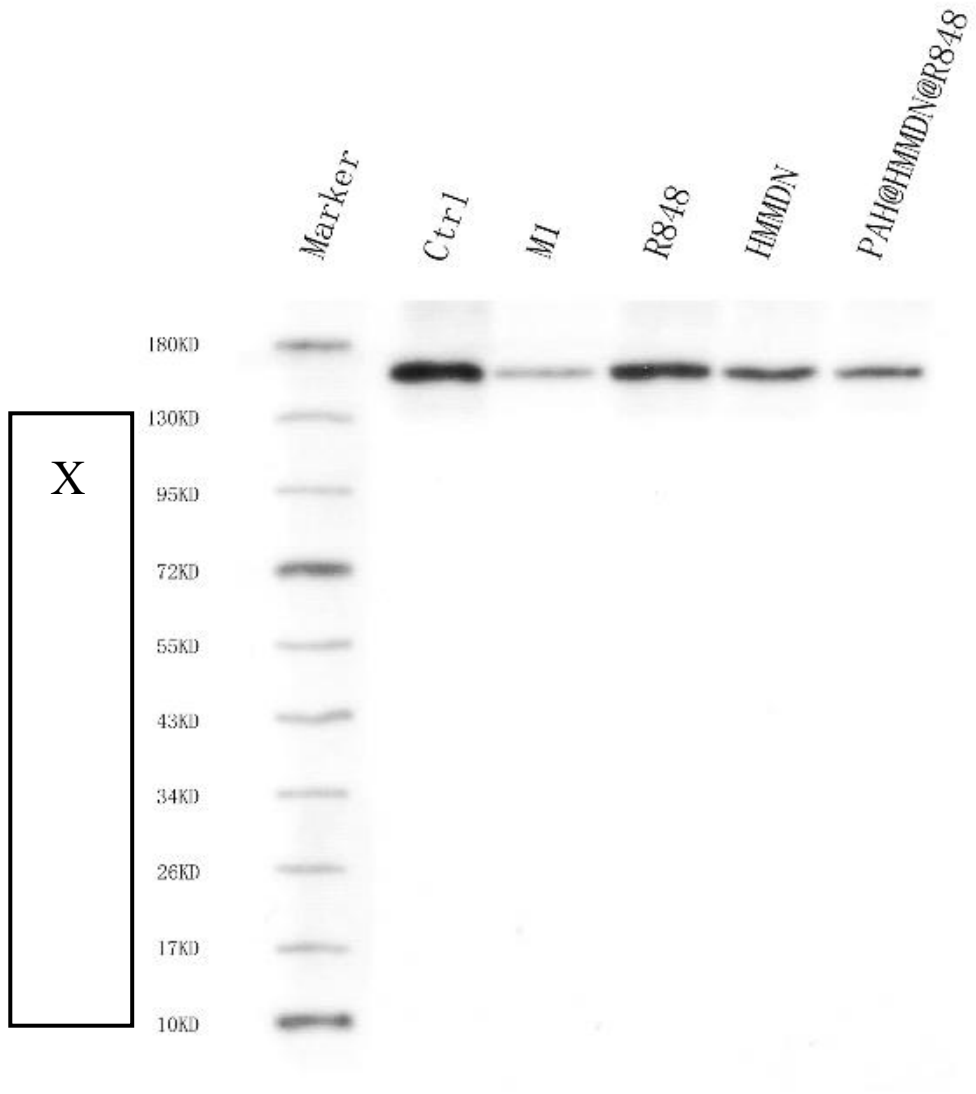

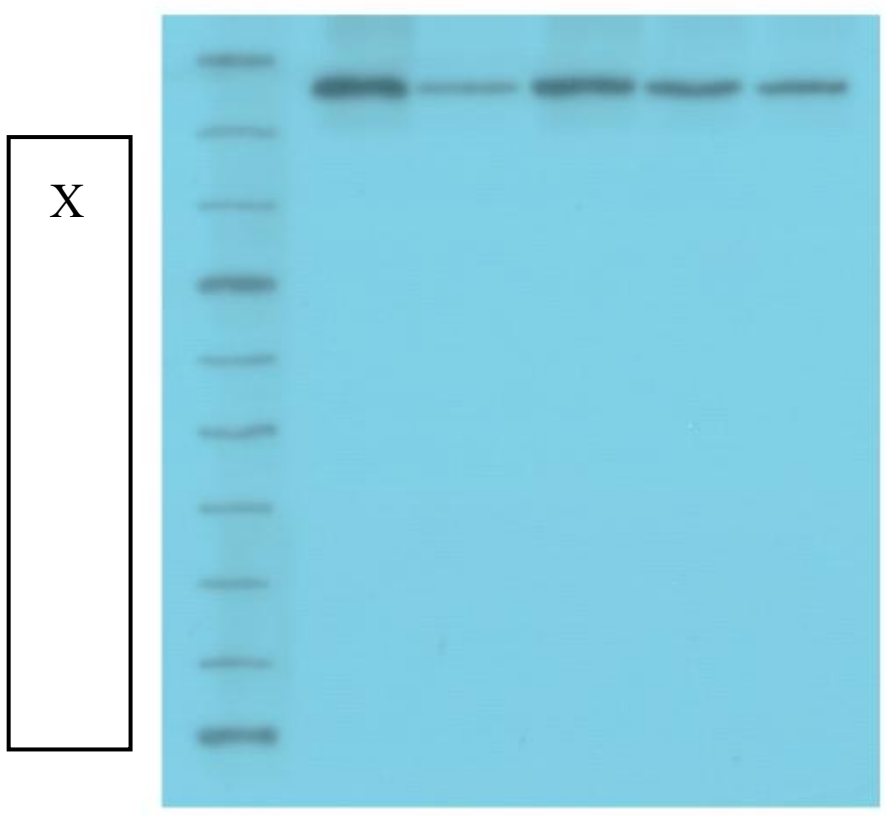

GADPH（FigS2A、FigS3B）

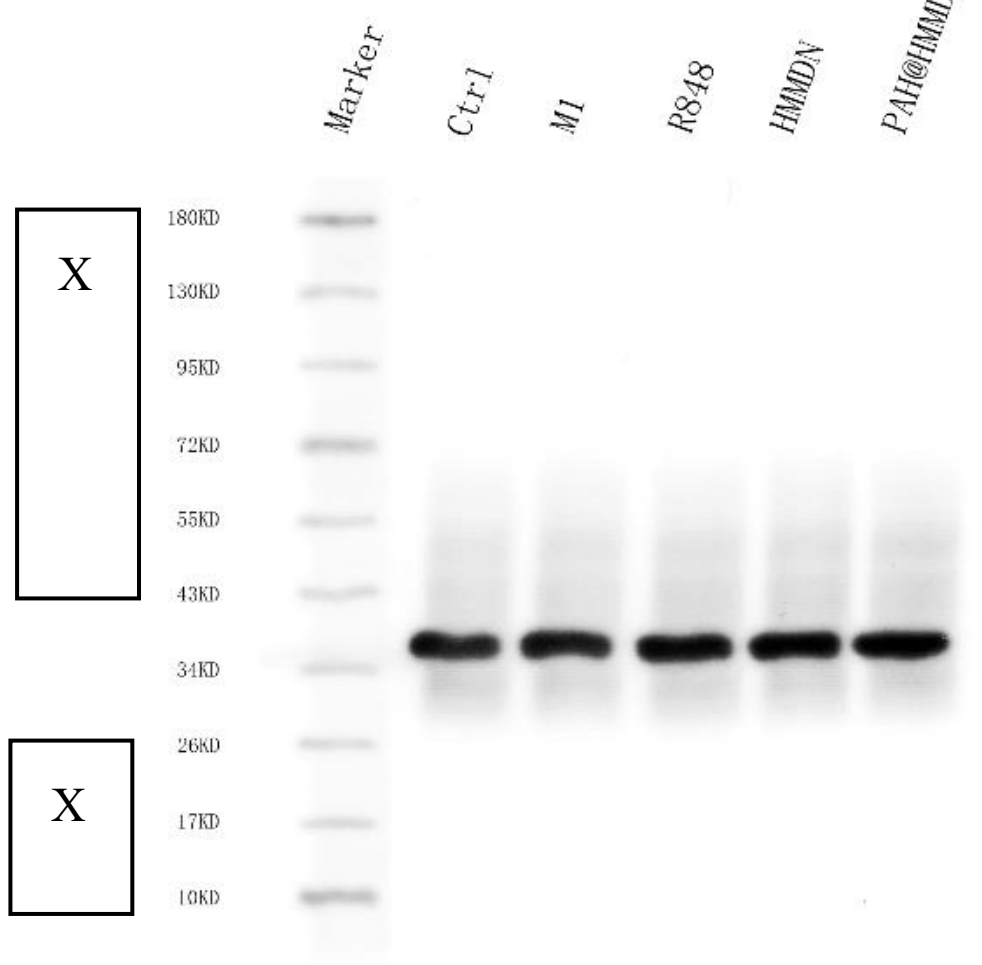

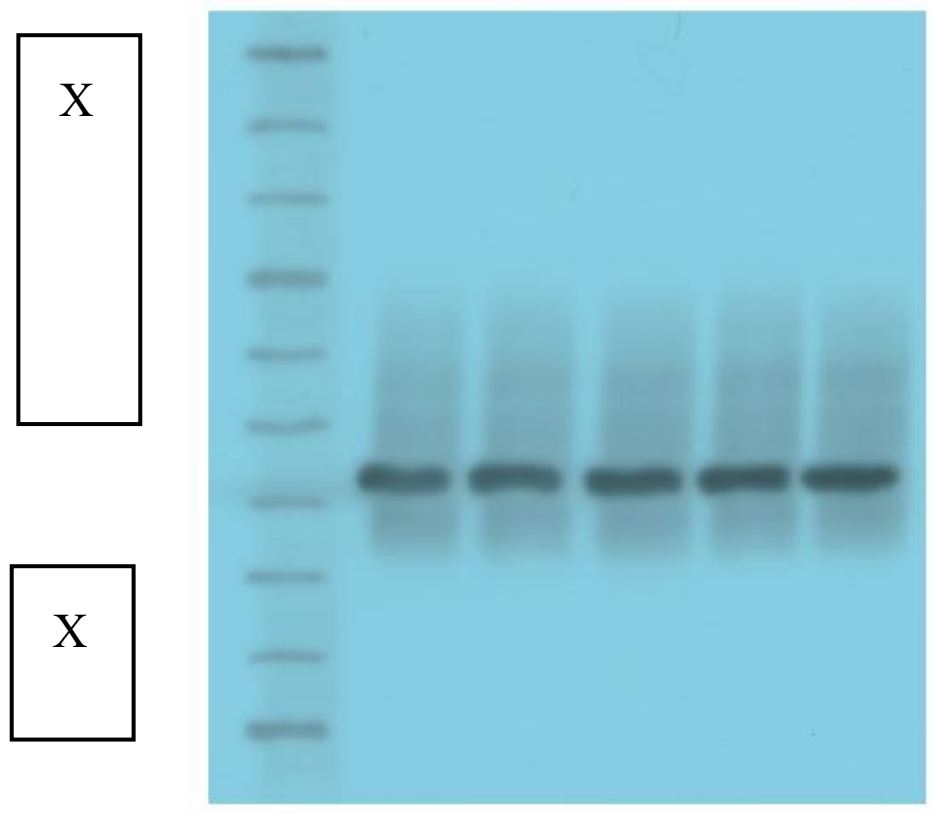

iNOS（FigS2A）

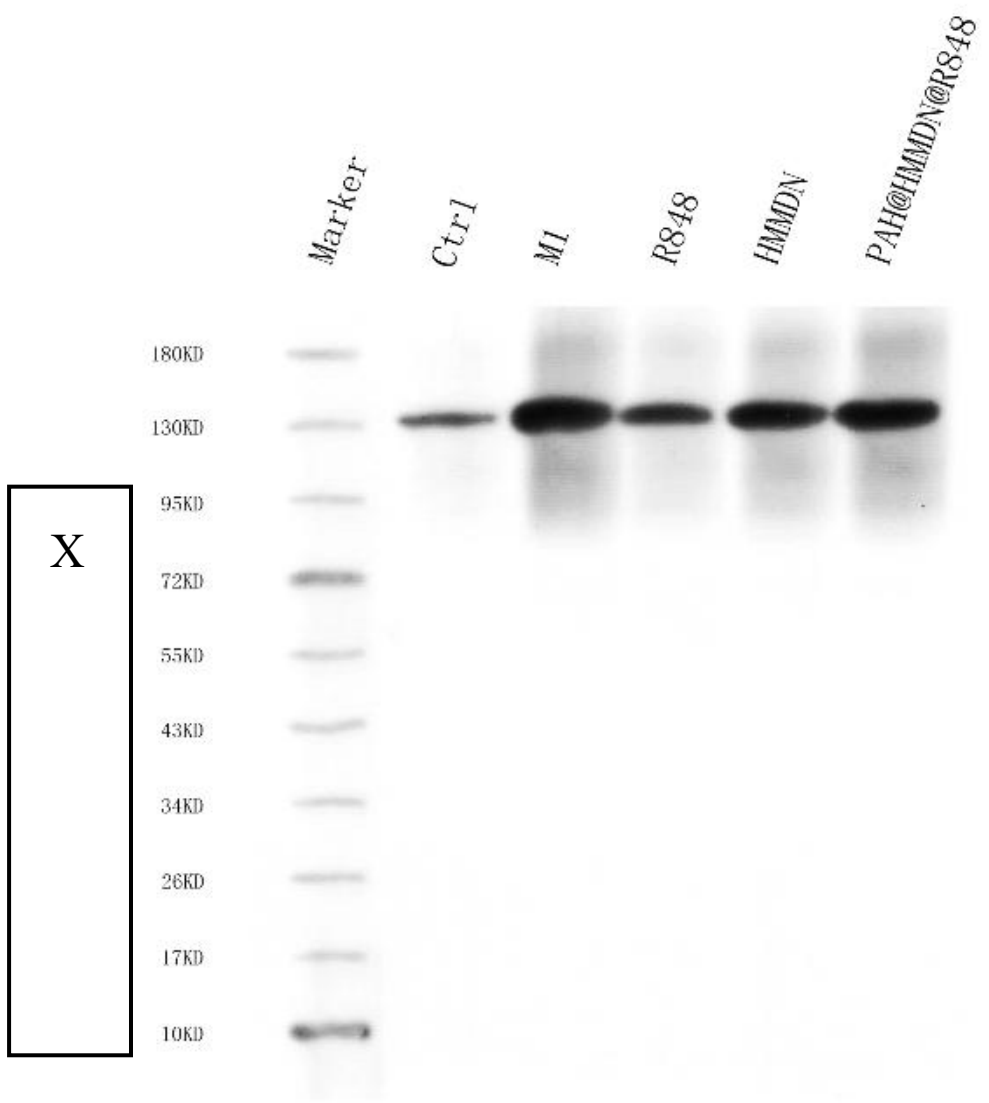
Marker
Ctrl
M1
R848
HMMDN
PAH@HMMDN@R848
180KD
130KD
95KD
72KD
55KD
43KD
34KD
26KD
17KD
10KD
X

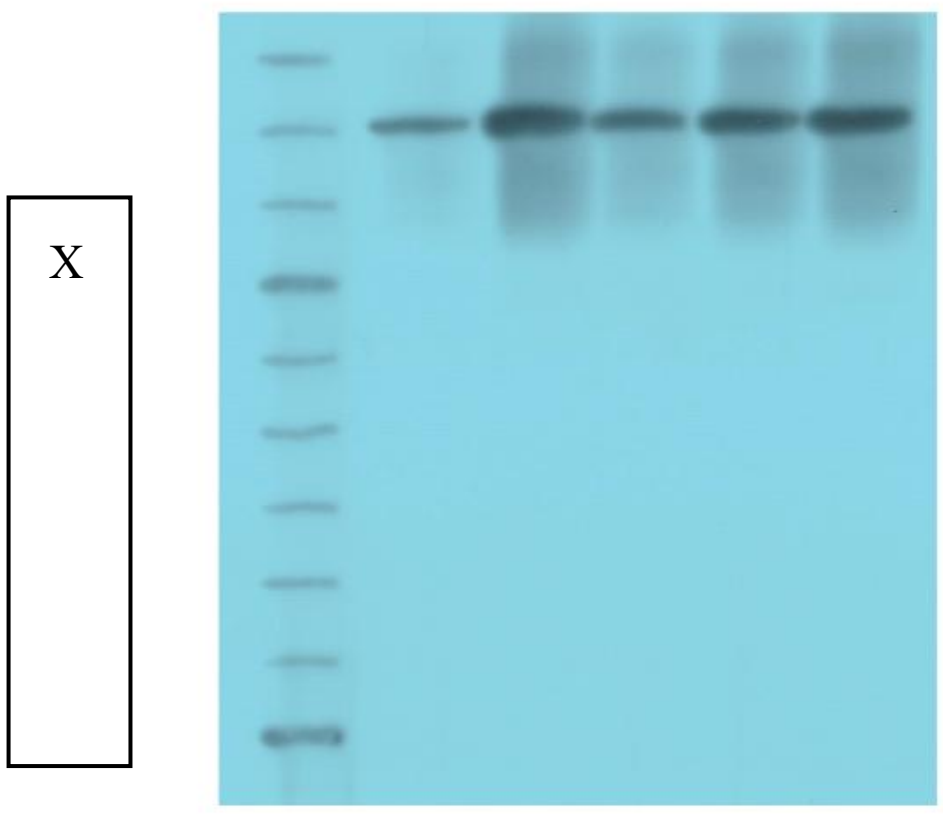
X

IRF3（FigS3B）

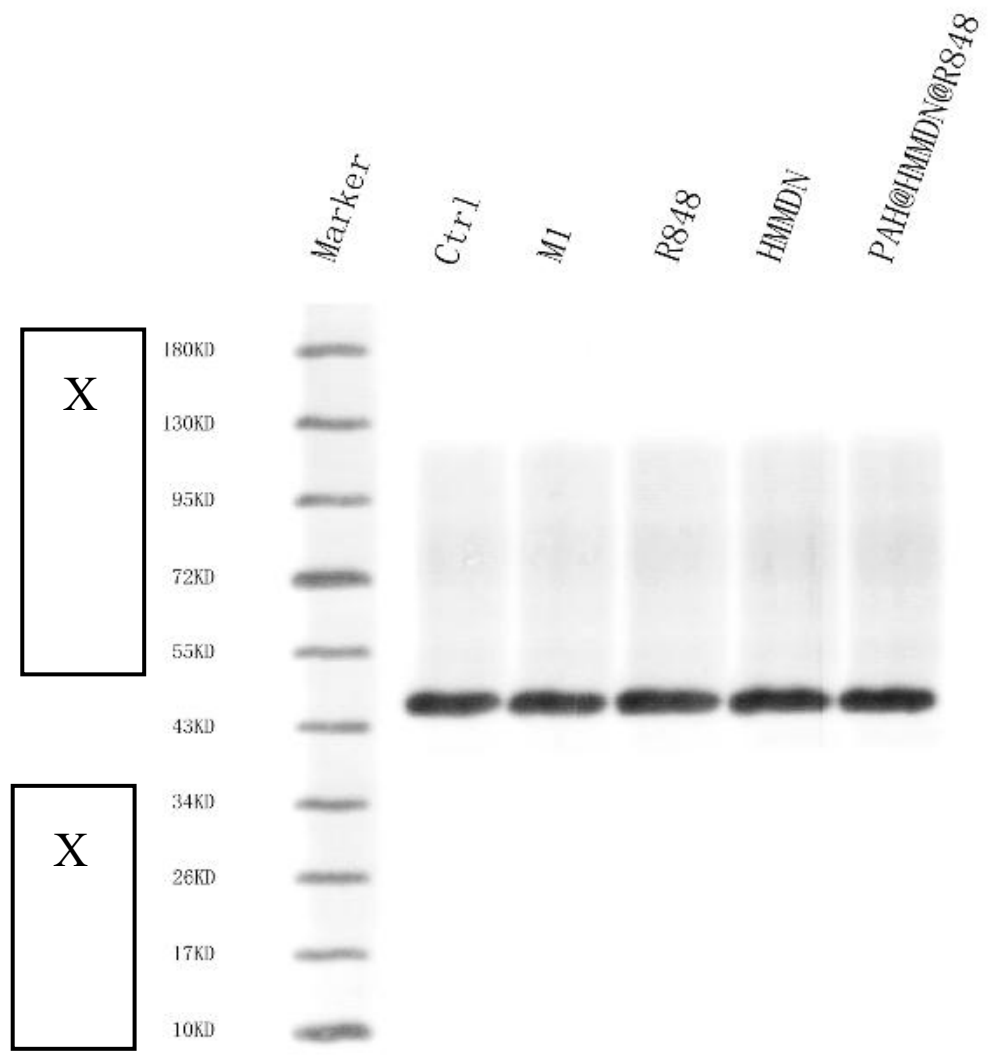
Marker
Ctrl
M1
R848
HMMDN
PAH@HMMDN@R848
180KD
130KD
95KD
72KD
55KD
43KD
34KD
26KD
17KD
10KD
X
X

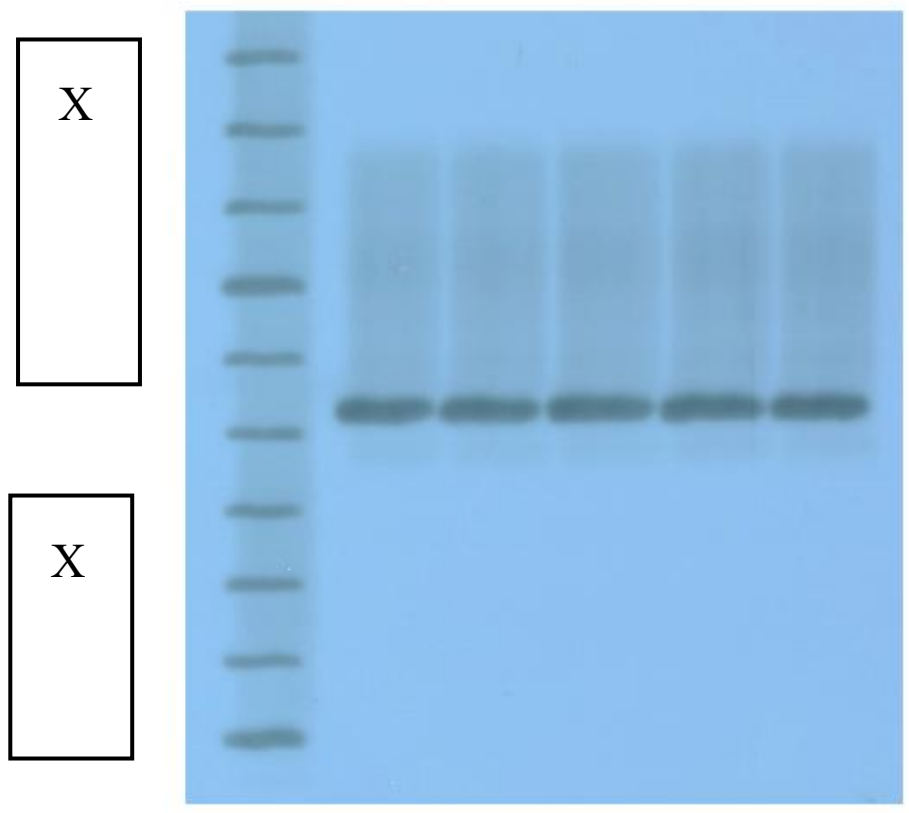
X
X

IκBα（FigS3A）

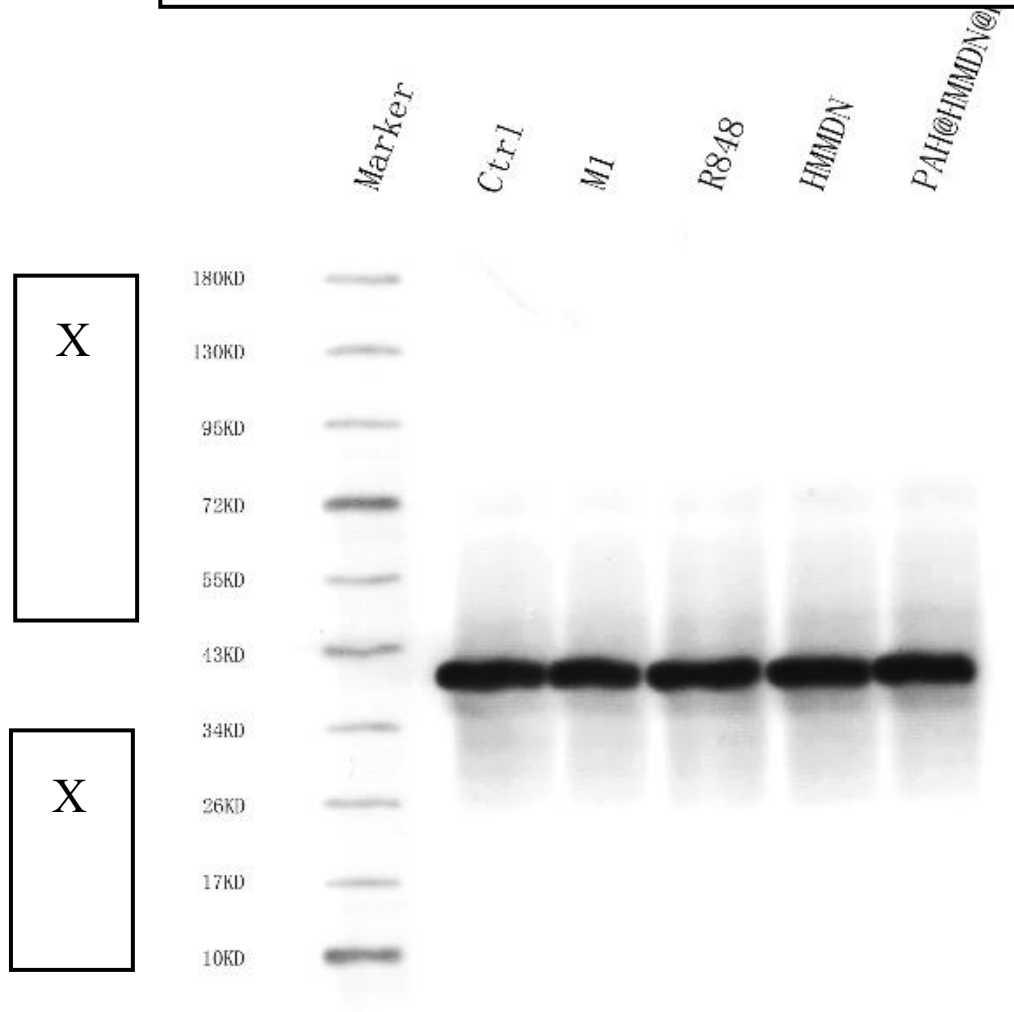
Marker
Ctrl
M1
R848
HMMDN
PAH@HMMDN@
180KD
130KD
95KD
72KD
55KD
43KD
34KD
26KD
17KD
10KD
X
X

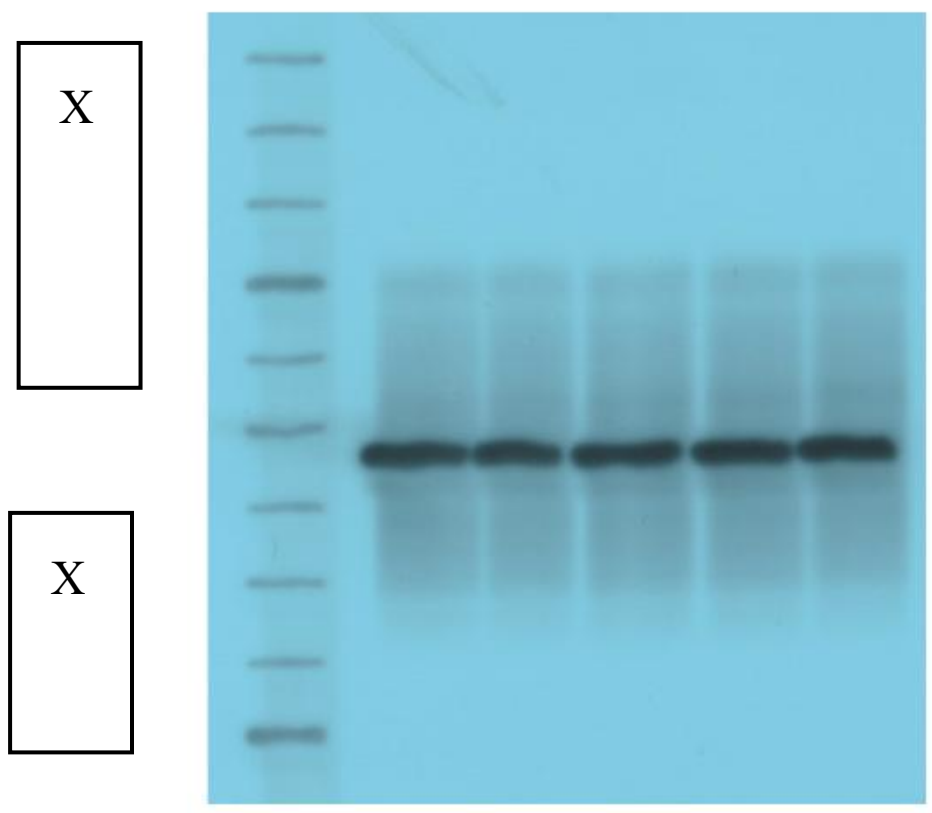
X
X

NFκB-p65（FigS3A）

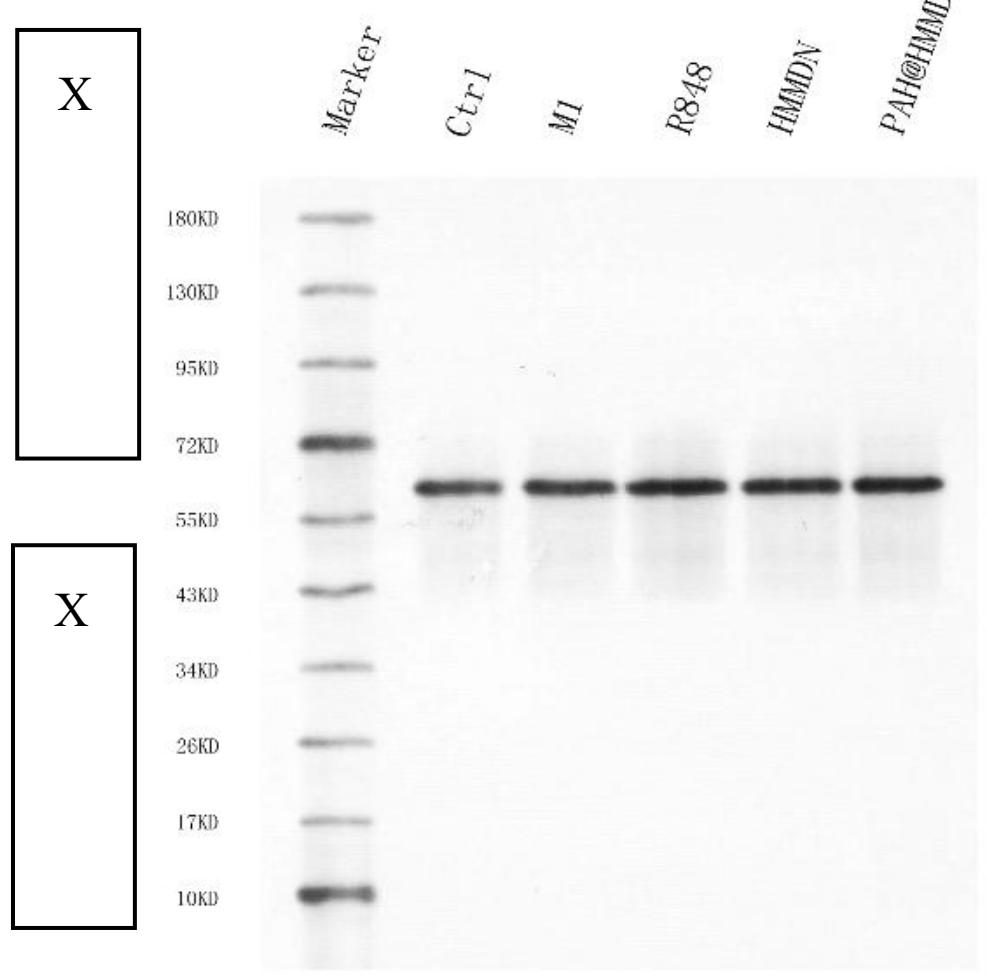

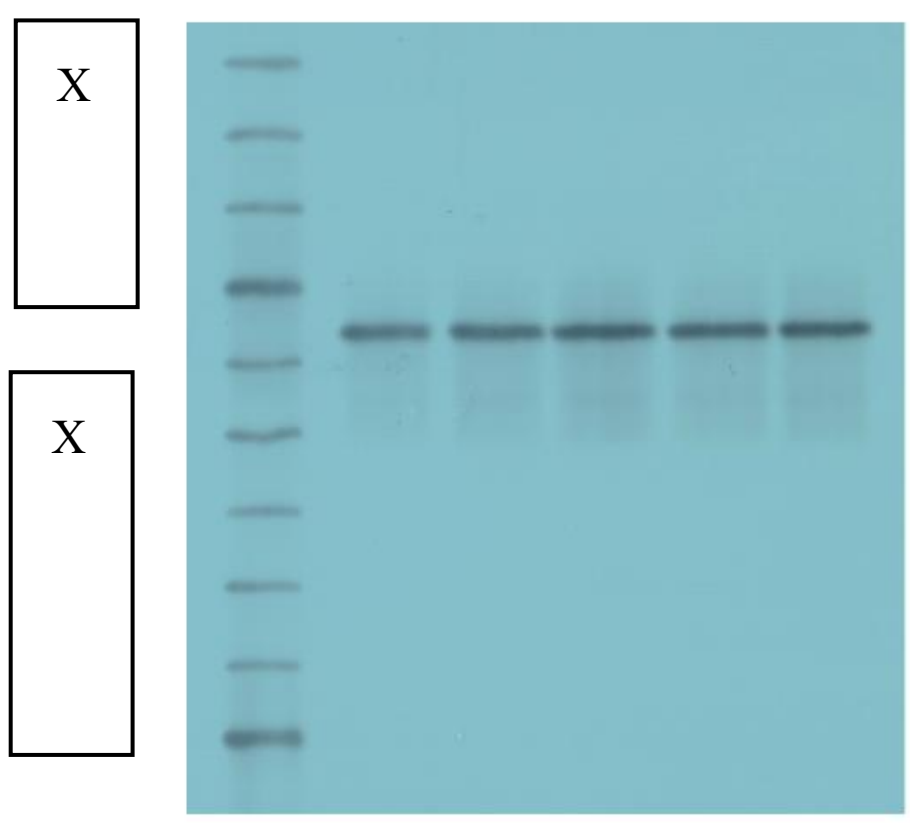

NFκB-p-p65（468）

（FigS3A）

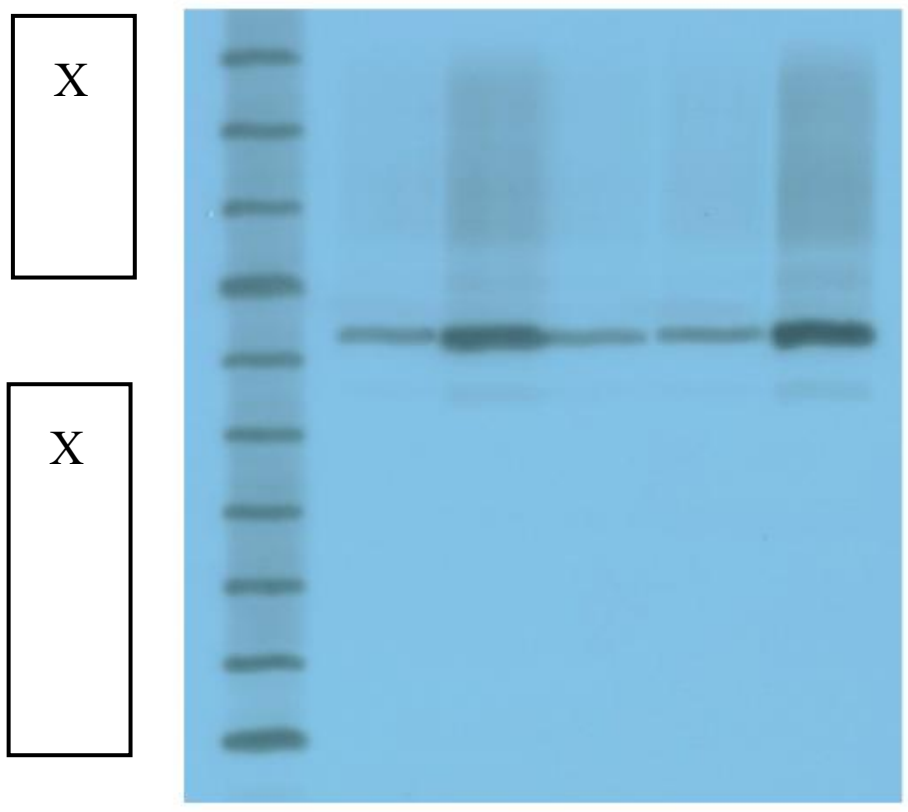

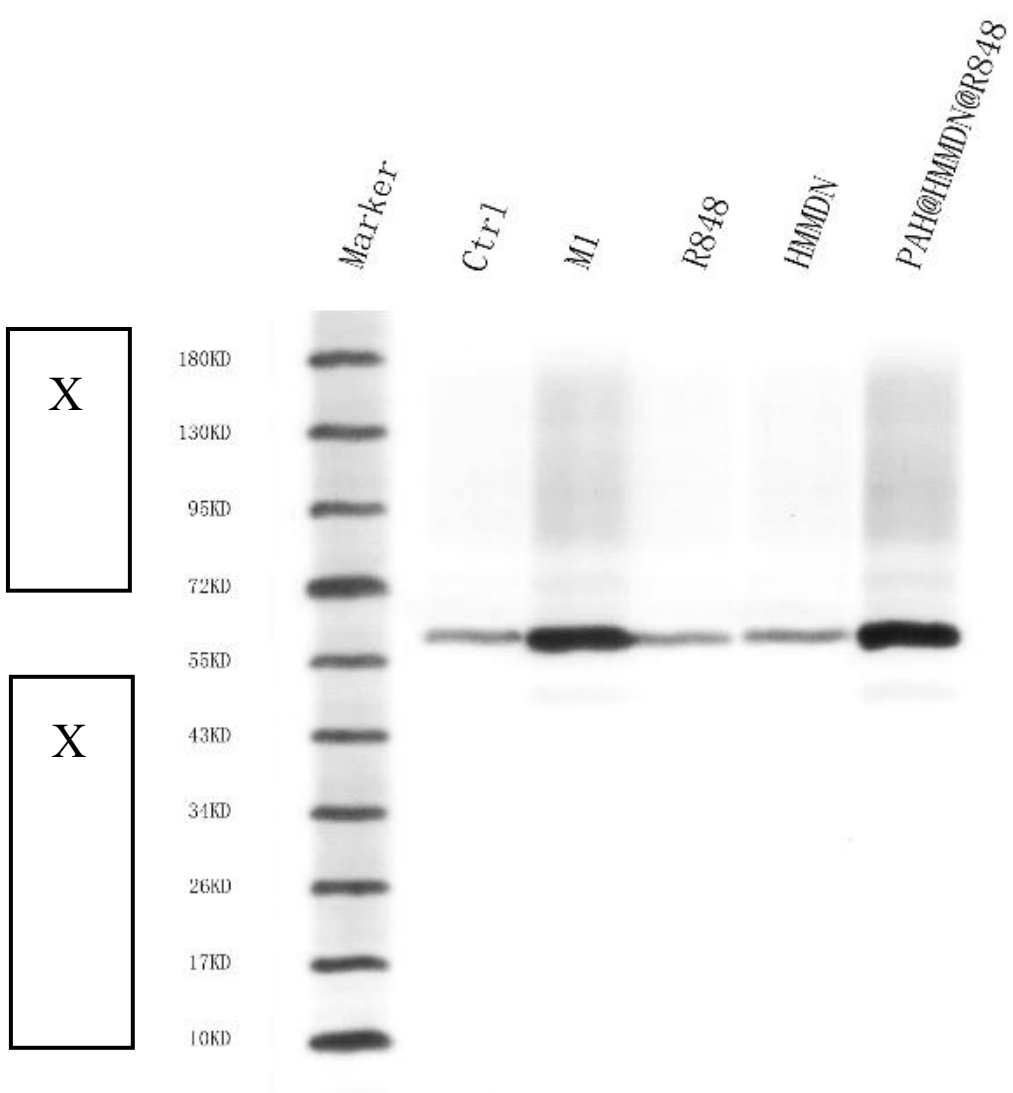

NF κ B-p-p65（536）

（FigS3A）

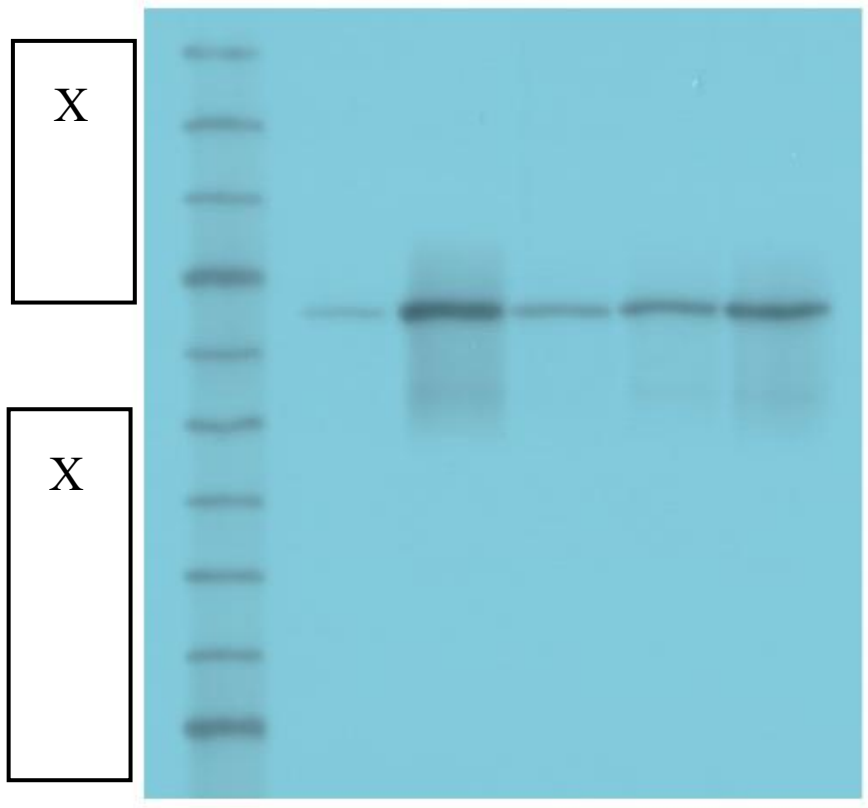

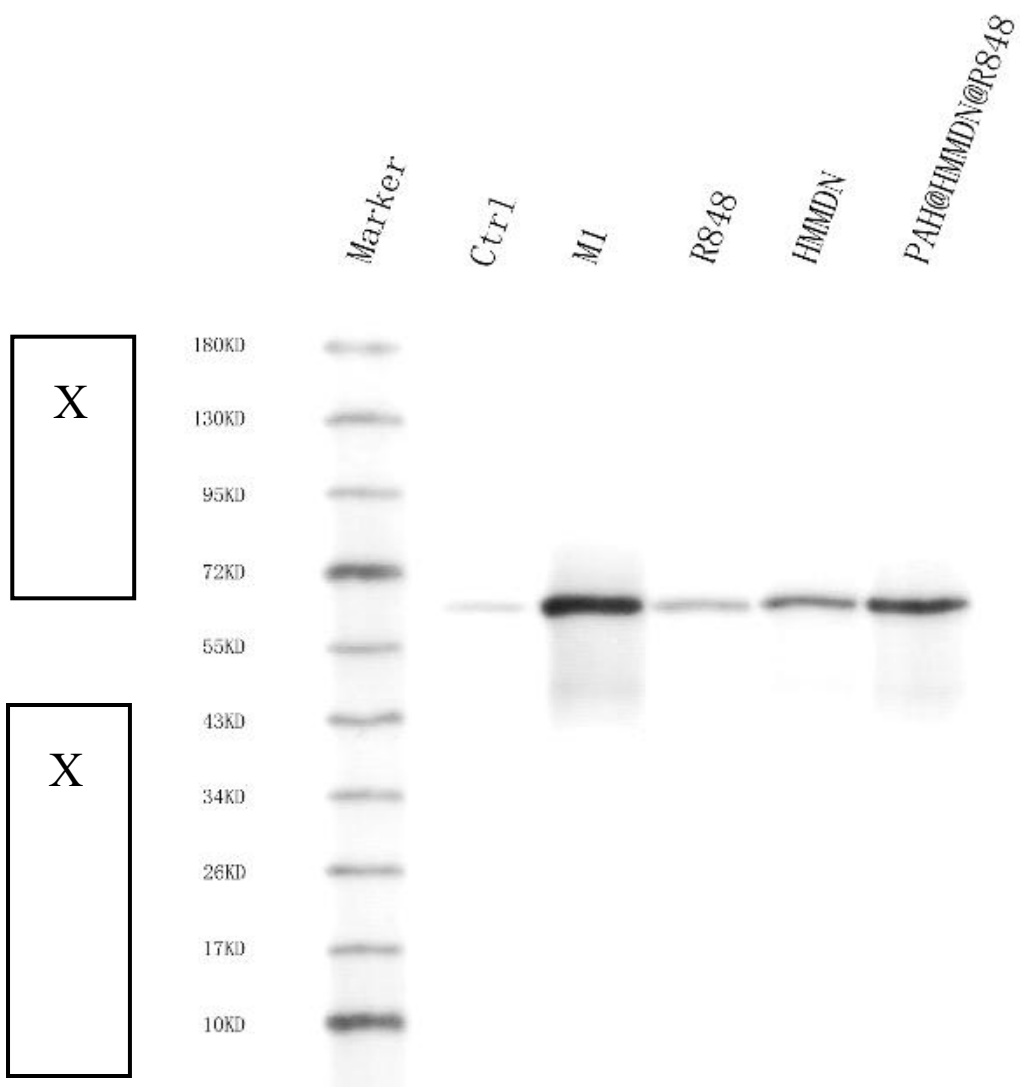

p-IRF3（FigS3B）

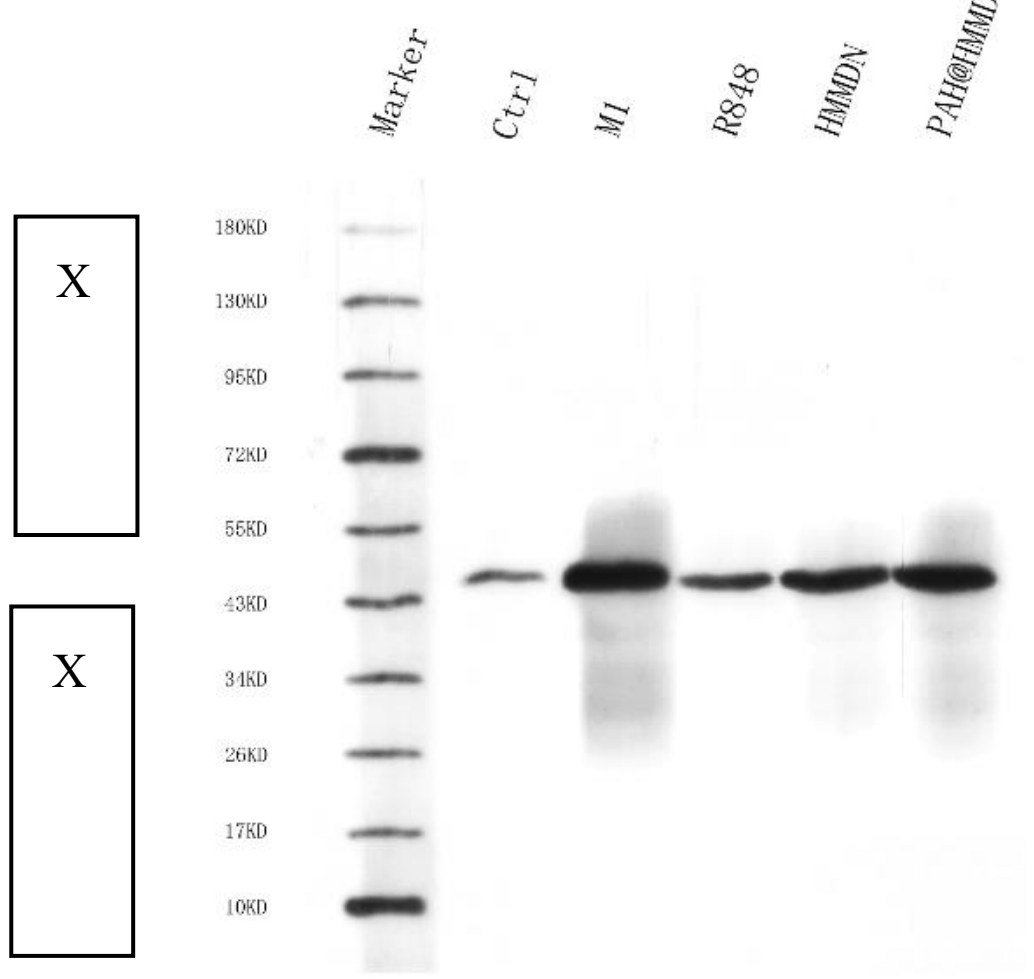

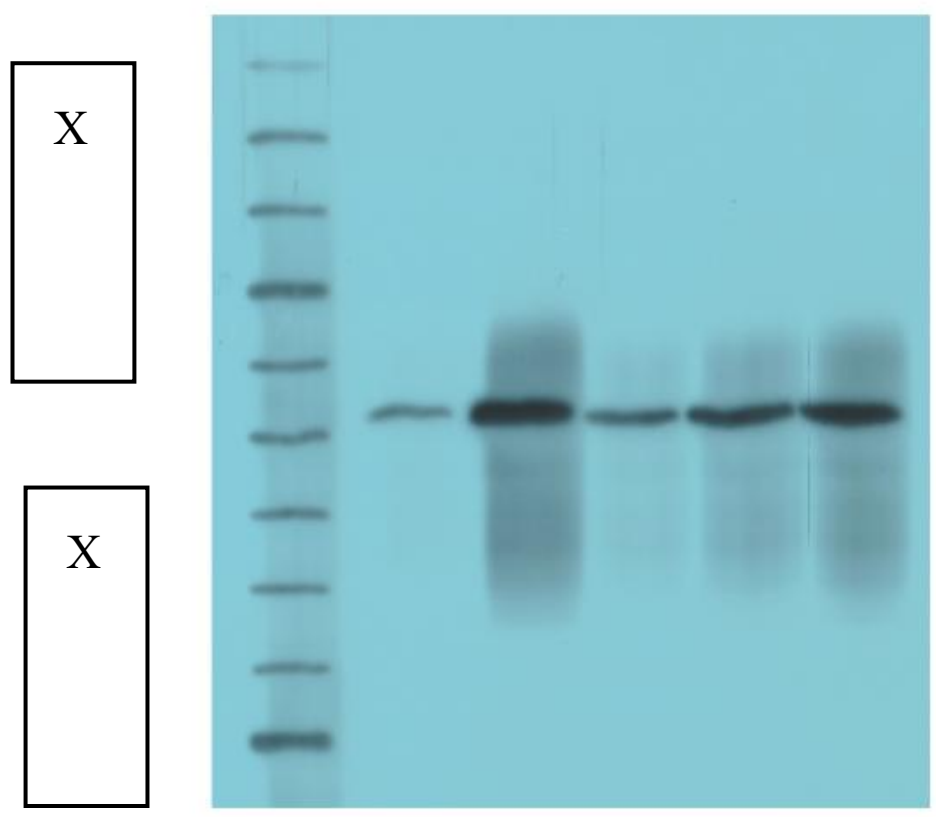

p-IκBα（FigS3A）

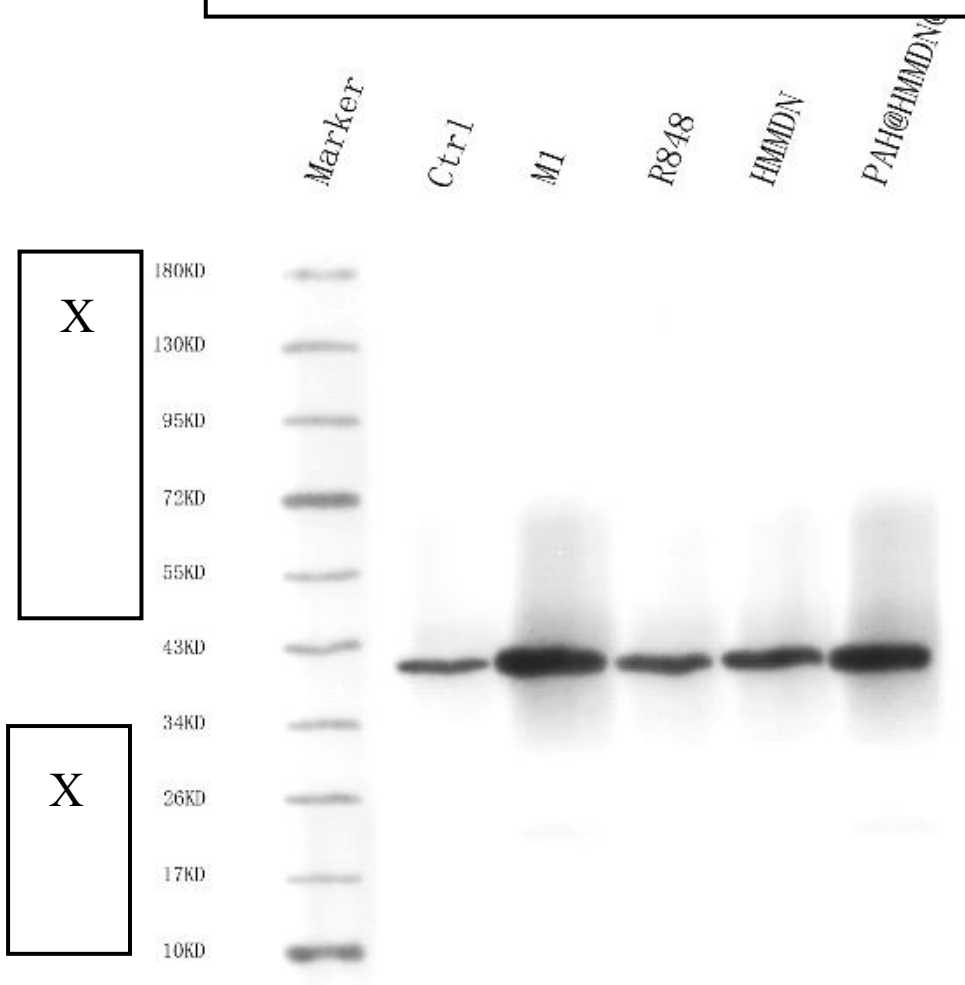

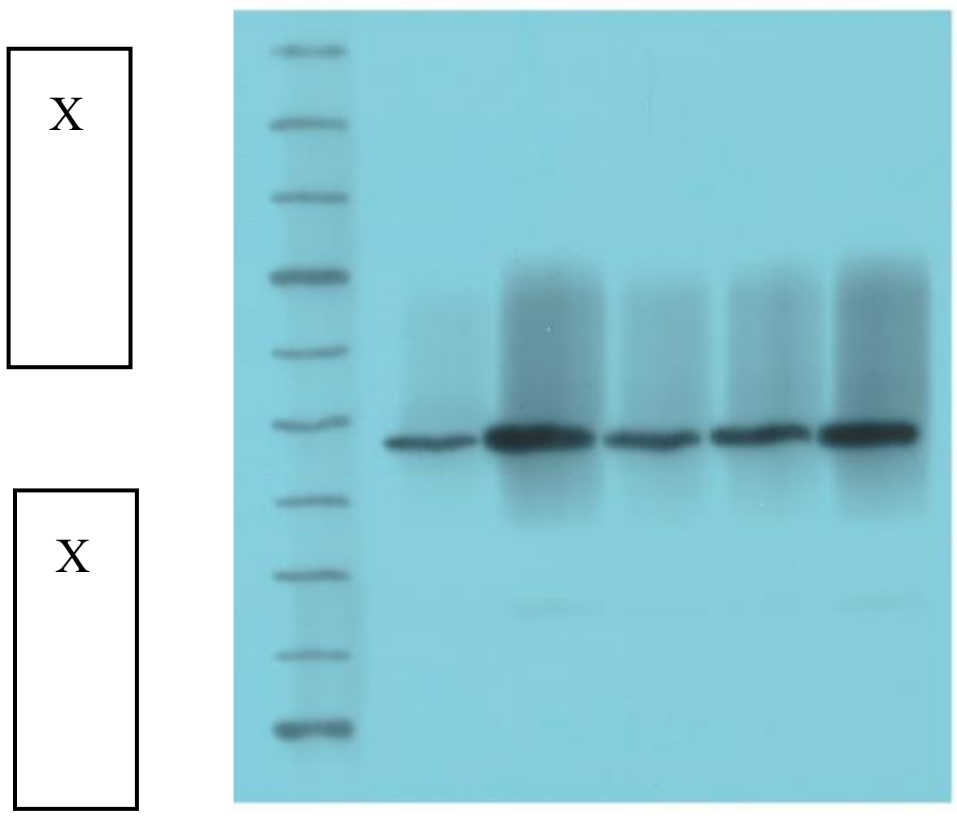

p-TBK1（FigS3B）

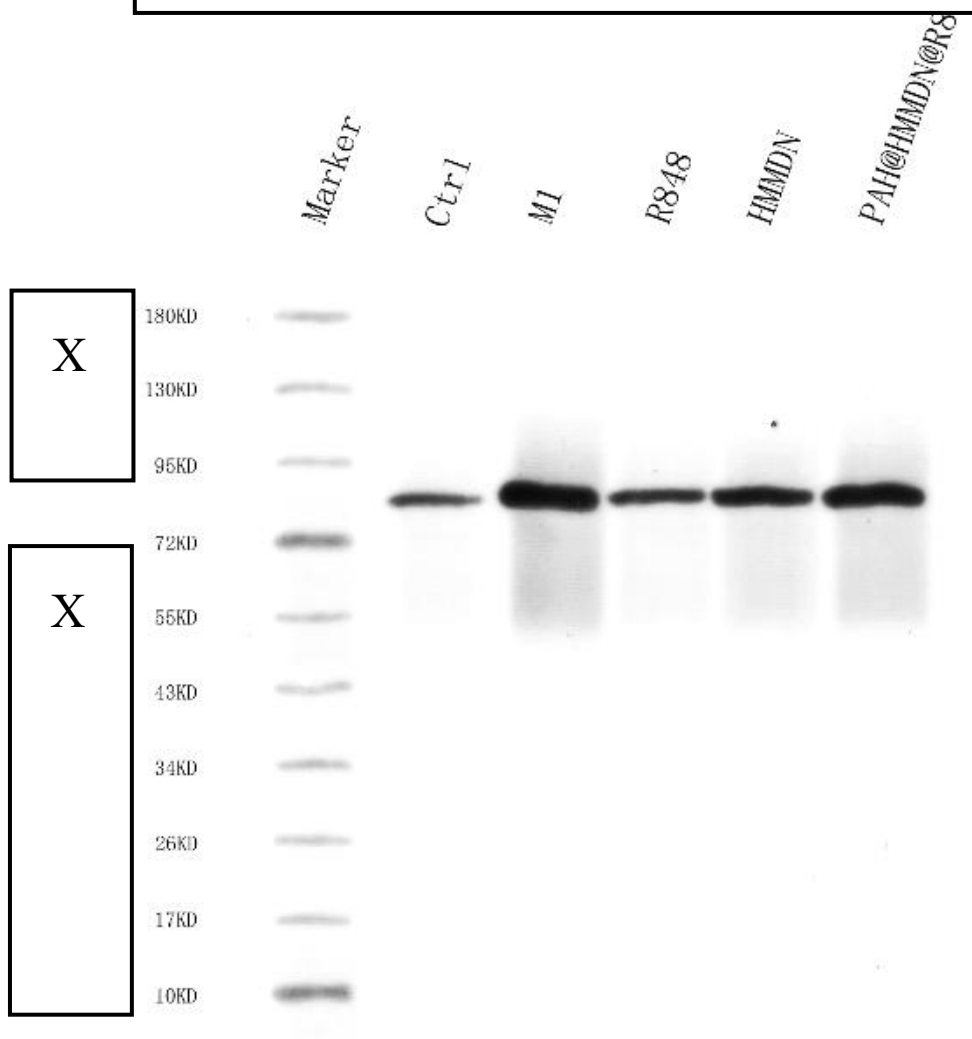

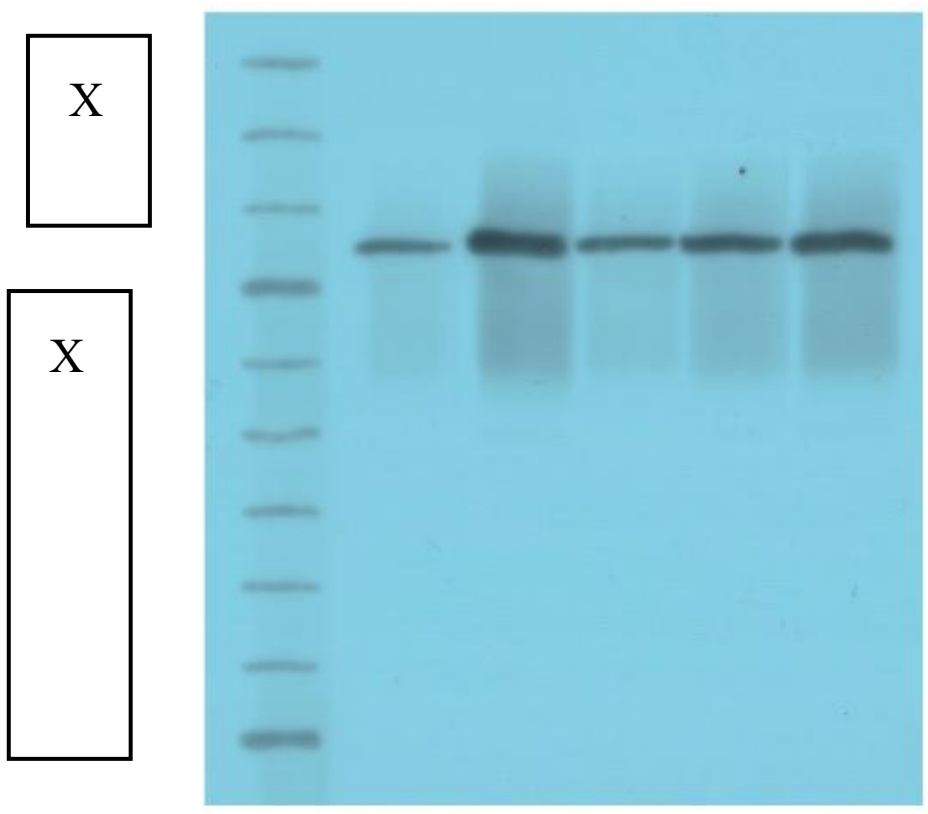

TBK1（FigS3B）

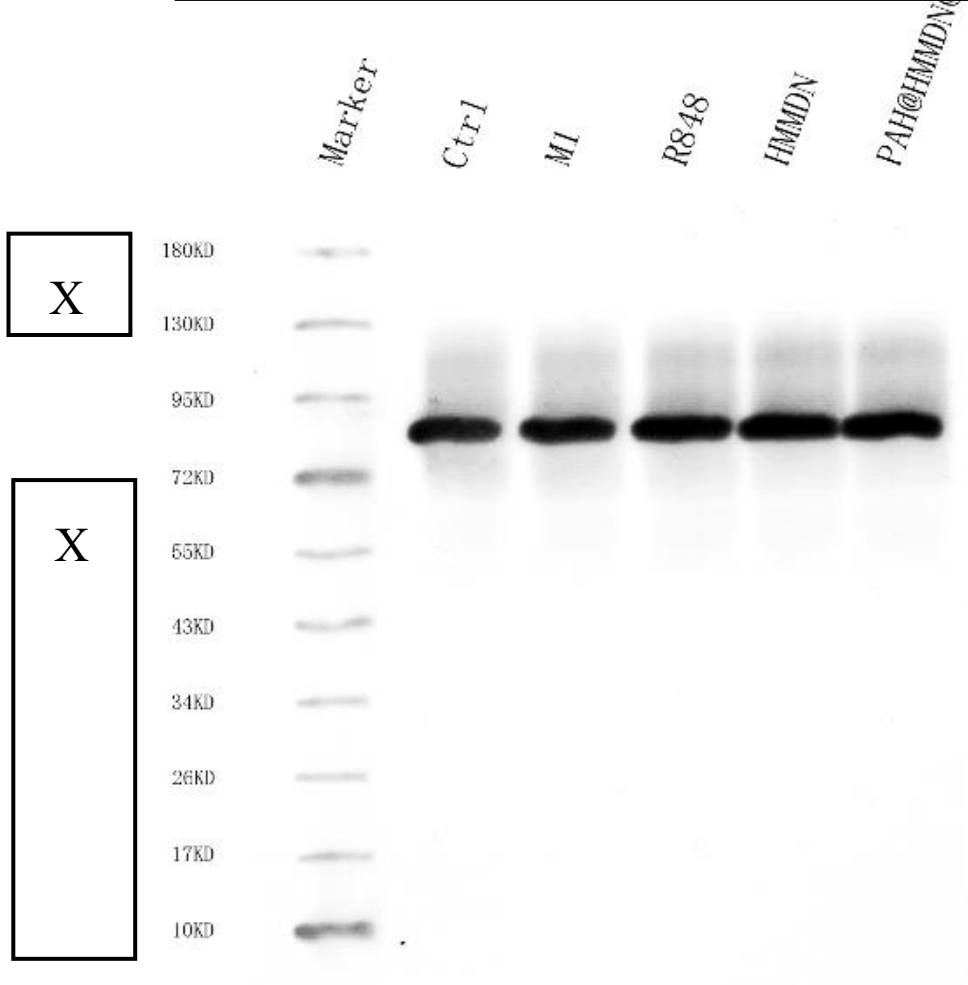
Marker
Ctrl
M1
R848
HMMDN
PAH@HMMDN
180KD
130KD
95KD
72KD
55KD
43KD
34KD
26KD
17KD
10KD
X
X

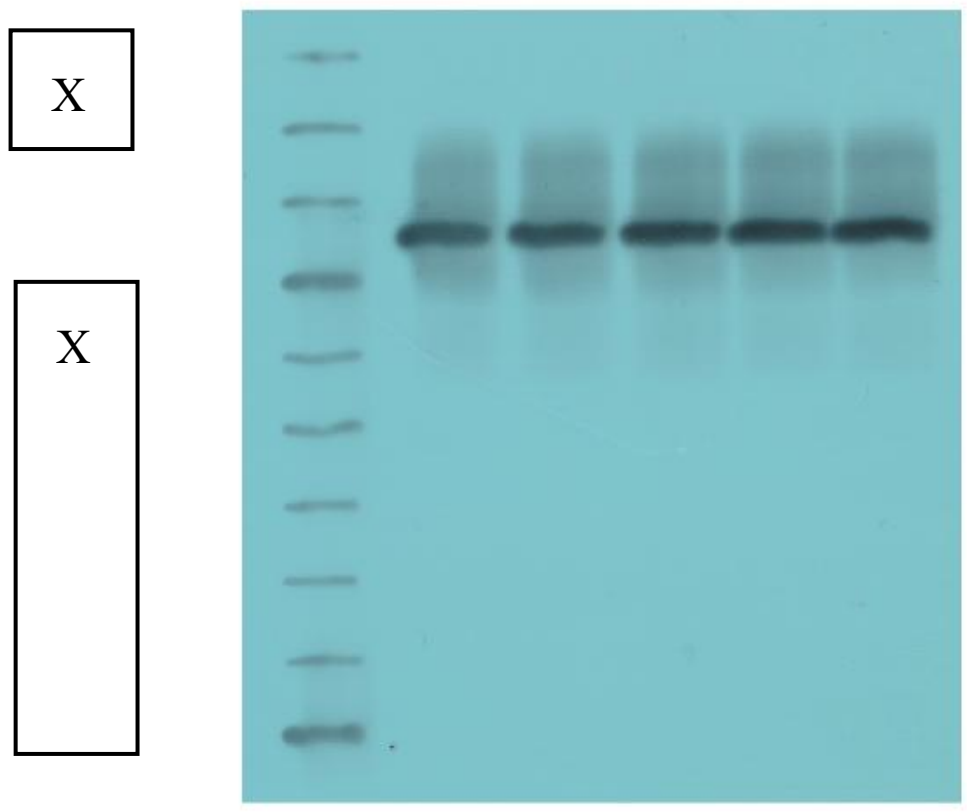
X
X
