# Supplementary material for: Reprogramming M2 macrophages via TLR7/8 agonist-loaded hollow MnO2 nanovehicles to suppress the progression of liver cancer
Source: PLoS One. 2026 Sep 18;21(9):e0358567. doi: 10.1371/journal.pone.0358567 (PMC13588417; doi:10.1371/journal.pone.0358567)
Supplement: Supplementary file 2 [file pone.0358567.s001.docx]

*PLOS ONE* Humane Endpoints Checklist

*PLOS ONE* manuscript number: ____**PONE-D-25-53955R2**___________________

**Complete the following if your study design includes death of a regulated animal as a likely outcome or planned experimental endpoint. Please also include all information in the Methods section of your manuscript.**

**ITEM 1.** **Describe whether humane endpoints* were used for all animals involved in the study.**

|  | **Recommendation** | **Section/Paragraph** |
| --- | --- | --- |
| **If humane endpoints* were used, report the following:** | | |
| **1** | **The specific criteria used to determine when animals should be euthanized** | Humane endpoint criteria were predefined in the IACUC-approved protocol (IACUC-20251215069) and strictly applied. Mice were euthanized immediately upon meeting any of the following criteria: (1) tumor diameter exceeding 1.5 cm in any direction or tumor volume exceeding 2,000 mm³; (2) body weight loss >20% of initial weight; (3) tumor ulceration, bleeding, or necrosis; (4) severe lethargy, persistent immobility, or inability to reach food or water; or (5) moribund condition as determined by the veterinarian. These criteria are consistent with internationally recognized humane endpoint standards for tumor-bearing rodents (National Research Council, 2011; NC3Rs guidelines). |
| **2** | **Once animals reached endpoint criteria, the amount of time elapsed before euthanasia** | Any animal meeting the predefined endpoint criteria was euthanized within 30 minutes of observation. |
| **3** | **Whether any animals died before meeting criteria for euthanasia** | No animals died before meeting the predefined humane endpoint criteria during the study. All animals were euthanized at the experimental endpoint (day 15) or upon reaching humane criteria, whichever occurred first. |
| **If humane endpoints* were not used, report the following:** | | |
| **1** | **A scientific and ethical justification for the study design, including the reasons why humane endpoints could not be used, and discussion of alternatives that were considered but could not be used** |  |
| **2** | **Whether the institutional animal ethics committee specifically reviewed and approved the anticipated mortality in the study design** |  |

**ITEM 2.** **Include the following details of the study design and outcomes.**

|  | **Recommendation** | **Section/Paragraph** |
| --- | --- | --- |
| **1** | **The duration of the experiment** | Total study duration: 365 days (including 7-day quarantine/acclimation period, 16-day administration period, and 342-day post-study observation/analysis). The in vivo treatment phase lasted 16 days from the first intratumoral injection to euthanasia, as specified in the IACUC-approved protocol (IACUC-20251215069). |
| **2** | **The numbers of animals used, euthanized, and found dead (if any); the cause of death for all animals** | Total animals used: 12 BALB/c nude mice (6–8 weeks old, 18–22 g). Group allocation: PBS control (n=3), free R848 (n=3), HMMDN (n=3), PAH@HMMDN@R848 (n=3). All 12 animals were euthanized at the experimental endpoint (day 15) as per protocol. No animals were found dead during the study. All deaths were procedure-related euthanasia per IACUC-approved humane endpoints. |
| **3** | **How frequently animal health and behavior were monitored** | All animals were monitored daily throughout the study for general health status, including body weight, feeding behavior, water intake, mobility, grooming, and tumor appearance. Tumor growth was measured with calipers every other day starting from day 5 post-inoculation. |
| **4** | **All animal welfare considerations taken, including efforts to minimize suffering and distress, use of analgesics or anaesthetics, or special housing conditions** | All procedures were conducted in accordance with institutional and national guidelines for the care and use of laboratory animals (National Research Council, 2011) and reported in compliance with the ARRIVE guidelines (Kilkenny et al., 2010). Animals were housed under specific pathogen-free conditions with a 12 h light/dark cycle and free access to food and water. All mice were acclimatized for 7 days prior to the experiment, and all injections were performed under brief isoflurane anesthesia to minimize pain. Humane endpoints were predefined and strictly applied to prevent unnecessary suffering. Daily health monitoring ensured early detection of any signs of distress. At the end of the experiment, mice were euthanized by intraperitoneal injection of 1% sodium pentobarbital (0.2 mL/10 g body weight) followed by cervical dislocation. |
| **5** | **Any special training in animal care or handling provided for research staff** | All personnel involved in animal procedures received appropriate training in laboratory animal care and handling, including aseptic surgical techniques, anesthesia administration, euthanasia procedures, and humane endpoint recognition, in accordance with institutional requirements. |

***Definition of a humane endpoint**

A humane endpoint is an experimental endpoint at which animals are euthanized when they display early markers associated with death or poor prognosis of quality of life, or specific signs of severe suffering or distress. Humane endpoints are used as an alternative to allowing such conditions to continue or progress to death following the experimental intervention (“death as an endpoint”), or only euthanizing animals at the end of an experiment. Before a study begins, researchers define the practical observations or measurements that will be used during the study to recognize a humane endpoint, based on anticipated clinical, physiological, and behavioral signs. These may include, for instance, body temperature or weight changes, tumor size or appearance, abnormal behaviors, pathological changes, ruffled fur, reduced mobility, body posture, or expression of specific body fluid markers. Please see the NC3Rs guidelines for more information.

**ARRIVE Guidelines**

*PLOS ONE* encourages authors to follow the [Animal Research: Reporting of In Vivo Experiments (ARRIVE) guidelines](http://www.nc3rs.org.uk/arrive-guidelines) for all submissions describing laboratory-based animal research and to upload a completed [ARRIVE Guidelines Checklist](http://www.nc3rs.org.uk/sites/default/files/documents/Guidelines/NC3Rs%20ARRIVE%20Guidelines%20Checklist%20%28fillable%29.pdf) to be published as supporting information.
